# β-Myrcene/isobornyl methacrylate SG1 nitroxide-mediated controlled radical polymerization: synthesis and characterization of gradient, diblock and triblock copolymers†

**DOI:** 10.1039/c8ra09192g

**Published:** 2019-01-25

**Authors:** Adrien Métafiot, Lysandre Gagnon, Sébastien Pruvost, Pascal Hubert, Jean-François Gérard, Brigitte Defoort, Milan Marić

**Affiliations:** Department of Chemical Engineering, McGill University 3610 University St. Montreal H3A 0C5 Quebec Canada milan.maric@mcgill.ca adrien.metafiot2@mcgill.ca; Department of Mechanical Engineering, McGill University 817 Sherbrooke St. W. Montreal H3A 0C3 Quebec Canada; Ingénierie des Matériaux Polymères (IMP), CNRS UMR5223, INSA Lyon 17 Jean Capelle Avenue, 69621 Villeurbanne France; ArianeGroup Avenue du Général Niox, 33160 Saint-Médard-en-Jalles France

## Abstract

β-Myrcene (My), a natural 1,3-diene, and isobornyl methacrylate (IBOMA), from partially bio-based raw materials sources, were copolymerized by nitroxide-mediated polymerization (NMP) in bulk using the SG1-based BlocBuilder™ alkoxyamine functionalized with an *N*-succinimidyl ester group, NHS-BlocBuilder, at *T* = 100 °C with initial IBOMA molar feed compositions *f*_IBOMA,0_ = 0.10–0.90. Copolymer reactivity ratios were *r*_My_ = 1.90–2.16 and *r*_IBOMA_ = 0.02–0.07 using Fineman–Ross, Kelen–Tudos and non-linear least-squares fitting to the Mayo–Lewis terminal model and indicated the possibility of gradient My/IBOMA copolymers. A linear increase in molecular weight *versus* conversion and a low dispersity (*Đ* ≤ 1.41) were exhibited by My/IBOMA copolymerization with *f*_IBOMA,0_ ≤ 0.80. My-rich and IBOMA-rich copolymers were shown to have a high degree of chain-end fidelity by performing subsequent chain-extensions with IBOMA and/or My, and by ^31^P NMR analysis. The preparation by NMP of My/IBOMA thermoplastic elastomers (TPEs), mostly bio-sourced, was then attempted. IBOMA-My-IBOMA triblock copolymers containing a minor fraction of My or styrene (S) units in the outer hard segments (*M*_n_ = 51–95 kg mol^−1^, *Đ* = 1.91–2.23 and *F*_IBOMA_ = 0.28–0.36) were synthesized using SG1-terminated poly(ethylene-*stat*-butylene) dialkoxyamine. The micro-phase separation was suggested by the detection of two distinct *T*_g_s at about −60 °C and +180 °C and confirmed by atomic force microscopy (AFM). A plastic stress–strain behavior (stress at break *σ*_B_ = 3.90 ± 0.22 MPa, elongation at break *ε*_B_ = 490 ± 31%) associated to an upper service temperature of about 140 °C were also highlighted for these triblock polymers.

## Abstract 2

β-Myrcene (My), a natural 1,3-diene, and isobornyl methacrylate (IBOMA), from partially bio-based raw materials sources, were copolymerized by nitroxide-mediated polymerization (NMP) in bulk.

## Graphical abstract

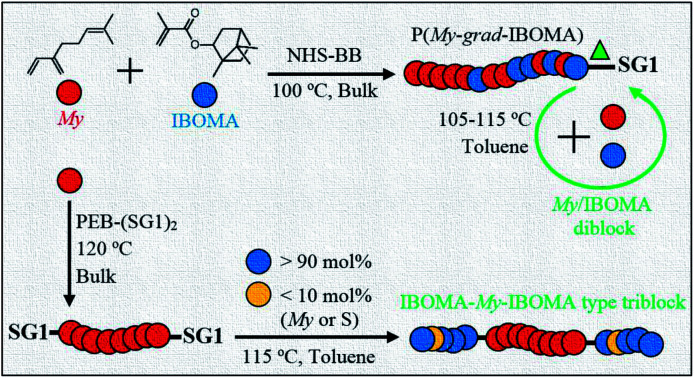

## Introduction

The community of polymer researchers has long desired to elaborate on polymerization techniques that couple the control of microstructure offered by living ionic polymerization^1–3^ with the simplicity of industrial implementation made possible by other processes such as free radical polymerization.^4^ Since the early 1980s, an easy-to-use synthetic technique now termed reversible-deactivation radical polymerization (RDRP)^5–9^ has been developed, encompassing several novel controlled/pseudo-living radical polymerizations and allowing the preparation of tailor-made macromolecules with precise and predetermined molar masses, compositions, topologies, and functionalities. Among them, nitroxide-mediated polymerization (NMP)^7,8,10^ is conceptually and practically the simplest of these systems. Based on a reversible termination mechanism between the propagating free radical and the nitroxide used as a controlling agent, NMP establishes a thermal dynamic equilibration between dormant macro-alkoxyamines capped by the nitroxide moiety and actively propagating macroradicals.^11^ At a minimum, only an alkoxyamine, available from commercial sources and easily functionalizable, and the monomer(s) are necessary to implement an NMP process initiated *via* a mono-component pathway (*i.e.* unimolecular initiator). NMP relies on the elimination of oxygen prior to polymerization as the main experimental constraint.^6,12^

The opportunity to prepare high-performance polymers by taking advantage of NMP's simplicity appears to be highly desirable from both practical and economical points of view.^13^ The synthesis of polymers having desirable mechanical properties such as a high tensile strength, a remarkable extensibility or a combination of both seems achievable by NMP. In particular, the development of styrenic-diene block copolymers, generally composed of a dispersed poly(styrene) (PS) or PS derivative phase in a continuous elastomer domain,^14,15^ looks specifically suited to the NMP process. Indeed, the NMP of conjugated dienes such as butadiene (B)^16,17^ and especially isoprene (I)^18^ was explored from the onset of the development of NMP, using 2,2,6,6-tetramethylpiperidinyl-1-oxy (TEMPO),^7^ the original first-generation stable free nitroxide. Well-tailored homopolymers and copolymers based on either B or I were produced as well,^19–23^ mediated by the 2,2,5-trimethyl-4-phenyl-3-azahexane-3-oxyl nitroxide (TIPNO),^19,24^ a more labile second-generation initiator. *N-tert*-Butyl-*N*-[1-diethylphosphono-(2,2-dimethylpropyl)]nitroxide (SG1)^25^ showed also its effectiveness to control the polymerization of I.^26–28^ Recently, we studied the well-controlled polymerization of β-myrcene,^29^ an acyclic monoterpene, initiated by the unimolecular SG1-based succinimidyl ester-functionalized BlocBuilder™ alkoxyamine called NHS-BlocBuilder,^30^ demonstrating once again the facility of the NMP process to synthesize elastomers based on 1,3-dienes with low dispersity (*Đ*) and substantial chain-end fidelity.

Well-tailored styrene (S)/I or B statistical, diblock and triblock polymers were thereby made by NMP.^19,31,32^ Surprisingly enough and to the best of our knowledge, no NMP-based styrenic-diene block copolymers were reported in the literature as possible alternative plastics with valuable mechanical properties or as thermoplastic elastomers (TPEs). This observation prompted us to synthesize NMP-based block copolymers based on an unusual but promising monomer coupling, namely β-myrcene (My)/isobornyl methacrylate (IBOMA).

A naturally occurring monoterpene with a highly active diene structure, β-myrcene (My, 7-methyl-3-methylene-octa-1,6-diene), typically exhibits chemistry similar to unsaturated hydrocarbons.^33–35^ Its polymerization leads to a bio-sourced P(My) elastomer exhibiting an expected sub-zero glass transition temperature (*T*_g_) of about −75 °C and a pseudo-plastic behavior.^36^ My-based TPEs were notably produced by anionic polymerization, making P(My) a possible elastomer substitute to well-known poly(butadiene) (PB) and poly(isoprene) (PI) rubbers. In 1983, S-My-S triblock copolymers, initiated by *sec*-butyllithium at 30 °C in benzene solution, were made by sequential monomer addition (number-average molecular weight *M*_n_ = 165–194 kg mol^−1^, final S molar fraction *F*_S_ = 0.23–0.58) and exhibited tensile strengths at break *σ*_B_ = 4.3–12.8 MPa and elongations at break *ε*_B_ = 670–1290%.^37,38^ Such triblocks exhibited the classical self-assembled morphologies in this case. More recently, Bolton *et al.* produced AMMS-My-AMMS (AMMS = α-methyl-*p*-methylstyrene) triblock polymers by sequential anionic polymerization,^39^ which showed ultimate tensile stress values ranging from 0.5 to 10.8 MPa and *ε*_B_ ranging from 525 to 1340%. Interestingly, the insertion of high *T*_g_ P(AMMS)s as outer hard blocks (*T*_g,P(AMMS)_ ∼ 180 °C) allowed the extension of the upper service temperature of these triblocks compared to that of traditional PS-based TPEs, which are limited to 90–100 °C.

Similarly, P(IBOMA), displaying a high *T*_g_ ∼ 190 °C,^40^ can be used as the rigid domain of block copolymer TPEs to provide strength, hardness and an improved performance at high temperature. This was illustrated by Yu and coworkers, who prepared IBOMA-B-IBOMA triblock copolymers initiated by the *m*-diisopropenylbenzene/*tert*-butyllithium diadduct,^41^ which exhibited excellent stress–strain properties and an upper service temperature of 160 °C. With the development of alkoxyamine unimolecular initiators and SG1 nitroxide in the late 1990s, the polymerization of IBOMA, a bulky hydrophobic methacrylate, is no longer a barrier by NMP. Control of methacrylate polymerizations was accomplished by Charleux and coworkers *via* SG1-based initiators using a small fraction of co-monomer such as S^42,43^ or acrylonitrile^44^ (<10 mol%). A comprehensive review was released by Nicolas et *al.* about the most successful strategies directed toward the control of the NMP of methacrylic esters.^45^ The development of dedicated nitroxides and alkoxyamines has led to improving the NMP of methacrylic esters. Guillaneuf, Gigmes and coworkers focused their attention on the indolynoxyl radical 2,2-diphenyl-3-phenylimino-2,3-dihydroindol-1-yloxyl (DPAIO) nitroxide,^46^ and the design of indolinic nitroxides derived from DPAIO.^47^ The bulk methyl methacrylate (MMA) polymerization initiated by the corresponding DPAIO-based alkoxyamines at 85–100 °C showed a good control. Recently, a new series of alkoxyamines were designed by Asua *et al.* and allowed the well-controlled synthesis of poly(methacrylate)s at moderate temperatures.^48^

In the present study, we report first the copolymerization of My and IBOMA in bulk at 100 °C, initiated by the succinimidyl ester-terminated NHS-BlocBuilder alkoxyamine. The active feature of these NMP-based My/IBOMA copolymers, ideally capped by a SG1 nitroxide moiety, was then assessed quantitatively *via*^31^P NMR spectroscopy and qualitatively by chain-extensions with a fresh batch of IBOMA and/or My. After guaranteeing the efficient control of the SG1-mediated copolymerization of My with IBOMA, the second part of this investigation was focused on the preparation of P(My)-(SG1)_2_ macroinitiators using telechelic poly(ethylene-*stat*-butylene) initiator terminated with SG1 nitroxide groups^49^ and their subsequent chain-extension with IBOMA-rich/My or S mixtures. The phase behavior, the thermal behavior, notably along with viscoelastic properties, and the stress–strain properties of the resulting triblock copolymers were elucidated to emphasize the possibility to produce valuable diene-based block copolymers from largely sustainable feedstocks, in a simple way by NMP.

## Experimental

### Materials & methods

β-Myrcene (My, ≥ 90%), basic alumina (Al_2_O_3_, Brockmann, Type I, 150 mesh), calcium hydride (CaH_2_, 90–95% reagent grade), thiophenol (97%), and benzene (≥99%, ACS reagent) were purchased from Sigma-Aldrich and used as received. Toluene (≥99%), methanol (MeOH, ≥99%), methylene chloride (CH_2_Cl_2_, 99.9% certified ACS), chloroform (CHCl_3_, 99.8%), and tetrahydrofuran (THF, 99.9% HPLC grade) were obtained from Fisher Scientific and used as received. 2-Methyl-2-[*N-tert*-butyl-*N*-(1-diethoxyphosphoryl-2,2-dimethylpropyl)-aminoxy]-*N*-propionyloxysuccinimide, also known as NHS-BlocBuilder (NHS-BB), was prepared according to a published method^30^ from 2-(*tert*-butyl[1-(diethoxyphosphoryl)-2,2-dimethylpropyl]aminooxy)-2-methylpropionic acid, also known as MAMA SG1 (BlocBuilder-MA™, (BB), 99%, provided by Arkema and used without further purification), *N*-hydroxysuccinimide (NHS, 98%, purchased from Aldrich and used as received), and *N*,*N*′-dicyclohexylcarbodiimide (DCC, 99%, purchased from Aldrich and used as received). Telechelic poly(ethylene-*stat*-butylene) difunctional initiator PEB-(SG1)_2_ terminated with *N-tert*-butyl-*N*-[1-diethylphosphono-(2,2-dimethylpropyl)]nitroxide (SG1) was produced relying on a published synthesis route,^49^ from hydroxyl terminated poly(ethylene-*stat*-butylene) (PEB-(OH)_2_, Kraton™ D2205) obtained from Kraton, acryloyl chloride (98%) acquired from Sigma-Aldrich and BlocBuilder™. Styrene (S, 99%) was obtained from Fisher Scientific and isobornyl methacrylate (IBOMA, Visiomer® Terra) was obtained from Evonik, and both S and IBOMA were purified by passing through a column of basic alumina mixed with 5 wt% calcium hydride and then stored in a sealed flask under a head of nitrogen in a refrigerator until needed. The deuterated chloroform (CDCl_3_, 99.8%) used as a solvent for nuclear magnetic resonance (NMR) was obtained from Cambridge Isotopes Laboratory. Diethyl phosphite (98%) was purchased from Sigma-Aldrich.

### My/IBOMA copolymerization by NMP

**Scheme 1:**
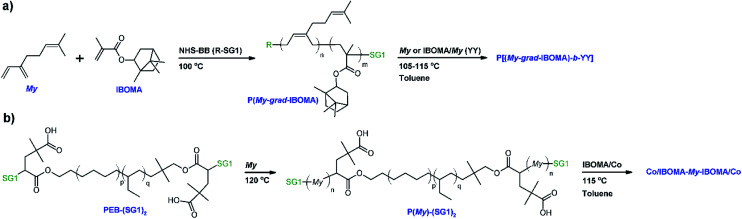
(a) My/IBOMA gradient copolymerization in bulk initiated by NHS-BB and subsequent My or IBOMA/My (∼93/7 mol%) chain-extension in toluene and (b) synthesis of P(My)-(SG1)_2_ from PEB-(SG1)_2_ difunctional initiator (*n* ≫ *p*, *q*) and subsequent IBOMA/Co chain-extension in toluene (Co = My or S co-monomer, 8–9 mol%).

The copolymerizations, depicted in Scheme 1a, were done in a 10 mL three-necked round-bottom glass flask equipped with a vertical reflux condenser (inserted in the middle neck), a thermal well, and a magnetic stir bar. Table 1 gives the formulations for the various My/IBOMA copolymerizations studied. For example, for the experiment My/IBOMA-50 (molar fraction of IBOMA in the initial feed *f*_IBOMA,0_ = 0.50), the reactor was sealed with rubber septa after the addition of NHS-BB (0.102 g, 0.213 mmol) and the magnetic stir bar. My (2.381 g, 17.478 mmol) and previously purified IBOMA (3.897 g, 17.529 mmol) were then injected into the reactor. The initial molar ratio of monomers and NHS-BB was calculated to give theoretically a copolymer sample with target number-average molecular weight *M*_n,theo_ = (*M*_My_*f*_My,0_ + *M*_IBOMA_*f*_IBOMA,0_) DP ∼ 30 kg mol^−1^ at complete overall conversion with DP = ([My]_0_ + [IBOMA]_0_)/[NHS-BB]_0_ = 164, the number average degree of polymerization. A mixture of ethylene glycol/distilled water (20/80 vol%) at a temperature of 3 °C was circulated (Fisher Scientific Isotemp 3016D digital refrigerated bath) through the condenser connected to one of the necks of the reactor to prevent any evaporative loss of the monomers. A purge of ultrapure nitrogen was then introduced to the reactor for 30 min to deoxygenate at room temperature the reactants prior to polymerization. After purging, the reactor was heated at a rate of about 10 °C min^−1^ to 100 °C with continuous nitrogen purge. The time at which the reactor temperature reached 90 °C was taken arbitrarily as the commencement of the reaction (*t* = 0 min). Samples were then taken from the reactor periodically by a syringe until the end of the experiments or until the samples became too viscous to withdraw. Reactions were then stopped by removing the reactor from the heating mantle and letting the contents cool down to room temperature, while under continuous nitrogen purge. For each sample withdrawn during the polymerization, the crude polymer was precipitated with excess methanol. After filtration and recovery, the precipitated polymer was dried at 50 °C under vacuum in the oven overnight to remove unreacted monomers. Samples were analyzed by nuclear magnetic resonance (NMR) and gel permeation chromatography (GPC). At the end of the experiment (*t* = 340 min), the overall conversion for My/IBOMA-50 was 52.7% (individual conversions: *X*_My_ = 75.2% and *X*_IBOMA_ = 30.2%) as determined by ^1^H NMR, with a number-average molecular-weight *M*_n,MHS_ = 9.4 kg mol^−1^ and a dispersity *Đ* = 1.28, as determined by GPC. The molar composition of IBOMA in the final copolymer was *F*_IBOMA_ = 0.43 according to ^1^H NMR spectroscopy. The exact same procedure was followed for all My/IBOMA copolymerizations. My/IBOMA copolymer characteristics can be found in Table 2.

**Table 1:** Formulations for My, IBOMA and My/IBOMA polymerizations performed in bulk or in toluene at *T* = 100–120 °C, initiated by NHS-BB and targeting *M*_n,theo_ = 30 kg mol^−1^ at *X* = 100%

ID^a^	[NHS-BB]_0_ (M)	[My]_0_ (M)	[IBOMA]_0_ (M)	*f*_My,0_	*T* (°C)	Solvent	*t* (min)
My/IBOMA-50-T120	0.030	2.49	2.51	0.50	120	—	240
My/IBOMA-70-T120	0.029	3.76	1.57	0.71	120	—	200
My/IBOMA-0-Tol	0.015	0	2.08	0	100	Toluene^b^	140
My/IBOMA-0	0.033	0	4.40	0	100	—	4^c^
My/IBOMA-10	0.032	0.45	4.04	0.10	100	—	108
My/IBOMA-20	0.032	0.91	3.73	0.20	100	—	240
My/IBOMA-30	0.031	1.45	3.33	0.30	100	—	300
My/IBOMA-40	0.031	1.94	2.92	0.40	100	—	280
My/IBOMA-50	0.031	2.51	2.52	0.50	100	—	340
My/IBOMA-60	0.028	3.07	2.10	0.59	100	—	380
My/IBOMA-70	0.029	3.70	1.61	0.70	100	—	480
My/IBOMA-80	0.028	4.46	1.08	0.81	100	—	380
My/IBOMA-90	0.027	5.09	0.55	0.90	100	—	400
My/IBOMA-100	0.026	5.80	0	1	100	—	500

a Experimental identification given by My/IBOMA-XX where XX refers to the rounded % initial molar fraction of My in the mixture (*f*_My,0_).

b 50 wt% of toluene in the initial feed.

c Highly viscous reaction medium after relatively short times for experiments having IBOMA-rich starting mixture.

**Table 2:** Molecular characterization and My selectivity at the end of the experiments and kinetic data of P(My), P(IBOMA) and P(My-grad-IBOMA) polymers initiated by NHS-BB and targeting *M*_n,theo_ = 30 kg mol^−1^ at *X* = 1.0

ID	*F*_My_ a	*X*_My_ b (%)	*X*_IBOMA_ b (%)	*X* b (%)	*M*_n,MHS_ c (kg mol^−1^)	*Đ* c	〈*k*_p_〉〈*K*〉^d^ (10^5^ s^−1^)	1,4-e (%)	1,2-e (%)
My/IBOMA-50-T120	0.52	97.1	73.5	85.3	16.1	1.48	6.4 ± 0.4	58.8	37.5
My/IBOMA-70-T120	0.70	98.8	82.9	94.2	13.7	1.71	12.7 ± 0.2	63.6	30.1
My/IBOMA-0-Tol	0	0	14.1	14.1	12.3	1.72	2.3 ± 0.8	—	—
My/IBOMA-0	0	0	38.4	38.4	27.1	1.75	—f	—	—
My/IBOMA-10	0.14	86.5	36.8	41.8	18.3	1.57	3.5 ± 0.2	—g	—g
My/IBOMA-20	0.51	81.5	11.1	25.2	8.6	1.38	0.9 ± 0.4	68.2	29.5
My/IBOMA-30	0.45	83.6	31.6	47.2	10.9	1.35	3.0 ± 0.2	61.9	36.0
My/IBOMA-40	0.64	66.2	16.6	36.4	6.5	1.36	0.9 ± 0.5	70.1	26.2
My/IBOMA-50	0.57	75.2	30.2	52.7	9.4	1.28	2.5 ± 0.3	65.9	30.5
My/IBOMA-60	0.83	77.5	15.3	52.0	7.3	1.26	2.7 ± 0.2	67.4	29.9
My/IBOMA-70	0.77	77.9	35.0	65.0	7.5	1.29	1.7 ± 0.6	71.3	24.9
My/IBOMA-80	0.81	57.8	56.0	57.5	7.4	1.41	1.8 ± 0.5	85.6	10.5
My/IBOMA-90	0.86	29.7	67.4	33.5	5.9	1.34	1.3 ± 0.7	86.4	8.5
My/IBOMA-100	1.0	39.4	0	39.4	5.0	1.32	1.4 ± 0.4	86.6	7.0

a Molar fraction of My in the copolymer (*F*_My_), as determined by ^1^H NMR in CDCl_3_ of the final dry sample (Fig. S5 in ESI for the spectral assignments).

b Individual monomer conversions *X*_My_ and *X*_IBOMA_, determined by ^1^H NMR in CDCl_3_. Overall conversion *X* = *X*_My_*f*_My,0_ + *X*_IBOMA_*f*_IBOMA,0_.

c *M*_n,GPC_ and *M*_w,GPC_ determined by GPC calibrated with PMMA standards in THF at 40 °C. *M*_n,MHS_ obtained from *M*_n,GPC_ and corrected using the Mark–Houwink relationship (further details in the Experimental section).

d 〈*k*_p_〉〈*K*〉 derived from the slopes 〈*k*_p_〉[P˙] taken from the semilogarithmic kinetic plots of ln((1 − *X*)^−1^) *versus* time in the linear region generally from 0 to 60 min (0 to 20 min for My/IBOMA-0-Tol and 0 to 120 min for My/IBOMA-80, My/IBOMA-90 and My/IBOMA-100; squared linear regression coefficient = *R*^2^ ≥ 0.91 for every experiment. The linear fits to the experimental data during the initial stages of the polymerizations are provided in the ESI, Fig. S3). 〈*k*_p_〉〈*K*〉's estimated from 〈*k*_p_〉[P˙] and *r* = [SG1]_0_/[NHS-BB]_0_ (eqn (5)). Error bars derived from the standard errors in the slope from the linear fits of ln((1 − *X*)^−1^) *versus* time.

e My regioselectivity determined by ^1^H NMR in CDCl_3_. 3,4-content% = 100 − 1,4-content% − 1,2-content% (Fig. S5 in ESI for further details).

f No kinetic study led due to the early “caking” (high viscosity) of the reaction medium.

g ^1^H NMR peaks could not be detected.

### Chain-extension of My/IBOMA copolymers with My, IBOMA and S

All chain-extensions from P(My-grad-IBOMA) macroinitiators were performed in a very similar setup to that used for the My/IBOMA copolymerizations with the use of a 10 mL reactor. The formulations can be found in Table 3B. As a brief illustration, My/IBOMA-82-IBOMA/My was synthesized using a gradient P(My-grad-IBOMA) copolymer (My/IBOMA-82, *F*_My_ = 0.82, *M*_n,MHS_ = 13.6 kg mol^−1^, *Đ* = 1.51, 0.367 g, 0.027 mmol) that was added to the reactor along with toluene (3.24 g), My (0.135 g, 0.991 mmol) and previously purified IBOMA (2.787 g, 12.530 mmol). The reaction medium was mixed and bubbled with ultrapure nitrogen for at least 30 min, then heated to 105 °C and allowed to react for 210 min while maintaining a N_2_ purge. *t* = 0 min was selected once the reaction medium reached 90 °C. Samples were drawn periodically *via* syringe at 0, 60, 120 and 210 min. The samples and the final diblock copolymer were precipitated in excess methanol and allowed to dry overnight in a vacuum oven at 50 °C. My/IBOMA-82-IBOMA/My exhibited *M*_n,MHS_ = 23.2 kg mol^−1^, *Đ* = 1.61 (GPC) and *F*_My_ = 0.49 (^1^H NMR). The results of the chain-extensions from P(My-grad-IBOMA)s are given in Table 3C.

Chain-extensions of (A) P(My-grad-IBOMA) macroinitiators (MI) with (B) IBOMA and/or My in 50 wt% toluene and (C) molecular characterization of the resulting chain-extended products

**Table:** 

(A) P(My-grad-IBOMA) macroinitiator^a^							
ID	*f*_My,0_	*F*_My,1_	LF^b^ (%)	*X*_1_ (%)	*M*_n,MHS,1_ (kg mol^−1^)	*M*_n,theo,*X*_1__ c (kg mol^−1^)	*Đ*_1_
My/IBOMA-44	0.30	0.44	74 ± 9	39.1	8.8	11.7	1.32
My/IBOMA-82	0.79	0.82	69 ± 3	64.2	13.6	19.3	1.51

a The index “1” is associated to the characteristics of the P(My-grad-IBOMA) macroinitiators (MI) whereas the index “2” refers to the features of the whole chain-extended diblock copolymer (MI + P(My) or P(IBOMA-*co*-My) segment added).

b Living molar fraction of MI chains capped by a SG1 group, measured by ^31^P NMR (Fig. S6 in ESI for the spectra). Standard deviation derived from the difference in macroinitiator *M*_n_ value between *M*_n,GPC_ and *M*_n,MHS_.

c Predicted *M*_n,MHS,1_ at *X*_1_ measured experimentally and calculated as follows: *M*_n,theo,*X*_1__ = (*X*_1_/100)*M*_n,theo,1_ with *M*_n,theo,1_ = 30 kg mol^−1^ at *X* = 100%.

d Targeted number-average molecular weight of the whole chain-extended diblock copolymer (MI block + extended block) at *X* = 100%.

e Predicted *M*_n,MHS,2_ of the whole chain-extended diblock copolymer (MI block + second block added) at *X*_2_, measured experimentally, and calculated as follows: *M*_n,theo,*X*_2__ = (*X*_2_/100)(*M*_n,theo,2_ − M_n,MHS,1_) + *M*_n,MHS,1_ (=predicted *M*_n_ of the second block added at *X*_2_ + experimental *M*_n,MHS_ of MI).

**Table:** 

(B) Formulation of chain-extension							
ID	[MI]_0_ (M)	[My]_0_ (M)	[IBOMA]_0_ (M)	[Toluene]_0_ (M)	*M*_n,theo_ d (kg mol^−1^)	*T* (°C)	*t* (min)
My/IBOMA-44-My	0.004	2.414	0	3.859	91.0	115	390
My/IBOMA-82-IBOMA/My	0.004	0.147	1.857	5.216	122.0	105	210

**Table:** 

(C) Chain-extended diblock copolymer^a^					
ID	*X*_2_ (%)	*F*_My,2_	*M*_n,MHS,2_ (kg mol^−1^)	*M*_n,theo,*X*_2__ e (kg mol^−1^)	*Đ*_2_
My/IBOMA-44-My	39.5	0.93	30.1	41.3	1.65
My/IBOMA-82-IBOMA/My	19.1	0.49	23.2	34.3	1.61

### Synthesis of Co/IBOMA-My-IBOMA/Co (Co = My or S co-monomer) triblock copolymers by NMP

A two-step process was implemented in a similar reactor apparatus used for the above-mentioned copolymerizations and chain-extensions: (1) My homopolymerization initiated by PEB-(SG1)_2_ dialkoxyamine; (2) IBOMA/Co chain-extension from P(My)-(SG1)_2_ macroinitiator. The entire set of formulations is given in Table 4. For example, the synthesis of S/IBOMA-My-IBOMA/S triblock polymer (Co = S), characterized then mechanically and rheologically, can be detailed. To the 50 mL reactor was added PEB-(SG1)_2_ difunctional initiator (*M*_n,GPC_ = 5.7 kg mol^−1^, *Đ* = 1.17, 0.912 g, 0.140 mmol) and My (21.005 g, 143.671 mmol) with magnetic stirring (experiment My-52, Table 4A). A purge of nitrogen was applied for 30 min. The reactor was then heated to 120 °C to commence polymerization. *t* = 0 min was selected once the reaction medium reached 100 °C. At *t* = 360 min, the polymerization was stopped, the polymer was precipitated in excess methanol and dried overnight in a vacuum oven at 50 °C. ^1^H NMR was performed to ensure the complete elimination of methanol and unreacted My in the dry polymer, and to determine the conversion (49.1%). P(My) synthesized exhibited *M*_n,MHS_ = 51.7 kg mol^−1^ and *Đ* = 1.53 as determined by GPC. The chain-extension was then done by reacting P(My)-(SG1)_2_ (1.50 g, 0.029 mmol), purified IBOMA (3.32 g, 14.94 mmol) and purified S (0.15 g, 1.42 mmol) in toluene (∼4.3 g) under continuous nitrogen flow and magnetic stirring for 3 h at 115 °C (experiment My-52-IBOMA/S, Table 4B). The resulting polymer sample (*M*_n,MHS_ = 78.1 g mol^−1^, *Đ* = 2.41) was precipitated in excess methanol and dried under vacuum at 50 °C overnight. Lastly, fractionation was performed using a methanol (non-solvent)/benzene (solvent) pair (2 cycles). The final fractionated triblock polymer exhibited *M*_n,MHS_ = 94.7 kg mol^−1^, *Đ* = 2.23, *F*_IBOMA_ = 0.34 and *F*_S_ = 0.07. Final Co/IBOMA-My-IBOMA/Co copolymer characteristics can be found in Table 4C.

Chain-extensions of (A) P(My)-(SG1)_2_ macroinitiators with (B) IBOMA/My and IBOMA/S mixtures at 115 °C in 50 wt% toluene and (C) features of the resulting triblock copolymers

**Table:** 

(A) P(My)-(SG1)_2_^a^							
ID	*t* (min)	*X*_My_ (%)	*M*_n,MHS,My_ (kg mol^−1^)	*M*_n,theo,*X*_My__ b (kg mol^−1^)	*Đ*_My_	1,4-c (%)	1,2-c (%)
My-35	290	33.4	35.2	47.9	1.45	91.7	3.2
My-52	360	49.1	51.7	68.6	1.53	88.2	5.0

a The indexes “My” and “CE” refer, respectively, to the final characteristics of P(My)-(SG1)_2_ and the whole chain-extended triblock copolymer.

b Predicted *M*_n,MHS,My_ at *X*_My_ measured experimentally and calculated as follows: *M*_n,theo,*X*_My__ = (*X*_My_/100)*M*_n,theo,My_ (*M*_n,theo,My_ = 143.4 and 139.8 kg mol^−1^ for experiments My-35 and My-52, respectively).

c See footnote “*e”* in Table 2.

d Targeted number-average molecular weight of the whole chain-extended triblock copolymer at *X*_CE_ = 100%.

e Predicted *M*_n,MHS,CE_ of the whole chain-extended triblock copolymer at *X*_CE_, measured experimentally, and calculated as follows: *M*_n,theo,*X*CE_ = (*X*_CE_/100)(*M*_n,theo,CE_ − *M*_n,MHS,My_) + *M*_n,MHS,My_.

**Table:** 

(B) Formulation of chain-extension								
ID	[P(My)-(SG1)_2_]_0_ (M)	[IBOMA]_0_ (M)	[My]_0_ (M)	[S]_0_ (M)	[Toluene]_0_ (M)	*M*_n,theo,CE_ d (kg mol^−1^)	*T* (°C)	*t* (min)
My-35-IBOMA/My	0.004	1.681	0.142	0	4.929	133.4	115	90
My-52-IBOMA/S	0.003	1.546	0	0.147	4.848	171.2	115	180

**Table:** 

(C) Chain-extended triblock copolymer^a^							
ID	*X*_CE_ (%)	*F*_My_	*F*_IBOMA_	*F*_S_	*M*_n,MHS,CE_ (kg mol^−1^)	*M*_n,theo,XCE_ e (kg mol^−1^)	*Đ*_CE_
My-35-IBOMA/My	28.6	0.72	0.28	0	51.3	63.3	1.91
My-52-IBOMA/S	59.0	0.59	0.36	0.05	94.7	122.2	2.23

### Characterization

Monomer conversion was determined with a Varian NMR Mercury spectrometer (^1^H NMR, 300 MHz, 32 scans) using CDCl_3_. In the case of a copolymerization between two monomers A and B, the overall monomer conversion *X* was calculated from formula (1):

1 *X* = *X*_A_*f*_A,0_ + *X*_B_*f*_B,0_

where *f*_A,0_ and *f*_B,0_ are the initial molar fractions of A and B, and *X*_A_ and *X*_B_ are the individual conversions of A and B, respectively. My conversion was calculated by comparing the integrated peaks of the aliphatic protons of the monomer (*δ* = 2.30–2.15 ppm, 4H), the aliphatic protons of the polymer (*δ* = 2.15–1.90 ppm, 8H) and the protons of the two methyl groups of both monomer and polymer (*δ* = 1.75–1.55 ppm, 6H). IBOMA conversion was obtained using the NMR peaks of the vinyl protons of the monomer (*δ* = 6.05 and 5.50 ppm, 2H) and the aromatic proton of both monomer and polymer (*δ* = 4.65–4.20 ppm, 1H). Styrene (S) conversion was determined using the vinyl protons of the monomer (*δ* = 6.80–6.70, 5.80–5.70 and 5.30–5.20 ppm, 3H) and the aromatic protons of both monomer and polymer (*δ* = 7.50–6.90 ppm, 5H). The same ^1^H NMR spectra (300 MHz Varian NMR Mercury spectrometer, CDCl_3_ eluent, 32 scans) allowed to determine the regioselectivity of the My repetitive units in the polymers by comparing the three integrated peaks at *δ* = 4.70–4.80 ppm (two vinyl protons of 3,4-addition and two vinyl protons corresponding to 1,2-addition), *δ* = 5.00–5.25 ppm (two olefinic protons corresponding to 1,4-addition, one olefinic proton corresponding to 1,2-addition and one olefinic proton from 3,4-addition) and *δ* = 5.30–5.50 ppm (one olefinic proton from 1,2-addition).^36,50^

Phosphorus nuclear magnetic resonance (^31^P NMR) was performed using a 5 mm diameter up NMR tube with 840 scans being processed in a 200 MHz Varian Gemini 2000 spectrometer operating at 81 MHz. 0.0352 g of My-rich My/IBOMA-82 (*M*_n,MHS_ = 13.6 kg mol^−1^) and 0.0676 g of IBOMA-rich My/IBOMA-44 (*M*_n,MHS_ = 8.8 kg mol^−1^) were characterized in CDCl_3_ with the addition of diethylphosphite as internal reference (0.0027 g and 0.0072 g respectively). To confirm similar relaxation rates between diethylphosphite and the copolymer samples, My/IBOMA-82 and My/IBOMA-44 were run under the exact same conditions with only one scan and no dummy scans (ss = 0). Slight differences (<3.2%) in integral values were measured between these spectra and the standard ones with multiple scans. Moderate *M*_n_ (∼10–20 kg mol^−1^) IBOMA/My copolymers were thus assumed to relax at the same rate compared to that of diethylphosphite.

The number-average molecular weights (*M*_n,GPC_) and the dispersities (*Đ* = *M*_w,GPC_/*M*_n,GPC_) were measured using gel permeation chromatography (GPC, Water Breeze, differential refractive index RI 2414 detector, 40 °C) with HPLC grade THF as the mobile phase (flow rate of 0.3 mL min^−1^). The GPC was equipped with 3 Waters Styragel® HR columns (HR1 with a molecular weight measurement range of 10^2^ to 5 × 10^3^ g mol^−1^, HR2 with a molecular weight measurement range of 5 × 10^2^ to 2 × 10^4^ g mol^−1^ and HR4 with a molecular weight measurement range of 5 × 10^3^ to 6 × 10^5^ g mol^−1^) and a guard column was used. *M*_n,GPC_ values were determined by calibration with 10 linear narrow molecular weight distribution P(MMA) standards (Varian Polymer Standards, molecular weights ranging from 875 to 1 677 000 g mol^−1^). The P(My) and P(IBOMA) contributions to *M*_n,GPC_ were converted using Mark–Houwink–Sakurada (MHS) coefficients (MHS parameters determined at 35 °C with THF eluent for P(MMA):^51^*K*_P(MMA)_ = 12.2 × 10^−5^ dL g^−1^ and *α*_P(MMA)_ = 0.690; MHS parameters determined at 25 °C with THF eluent for P(IBOMA):^52^*K*_P(IBOMA)_ = 3.8 × 10^−5^ dL g^−1^ and *α*_P(IBOMA)_ = 0.748; MHS parameters determined at 30 °C with THF eluent for P(My) containing 90 and 10 mol% of 1,4- and 3.4-content respectively:^53^*K*_P(My)_ = 7.5 × 10^−5^ dL g^−1^ and *α*_P(My)_ = 0.772). Therefore, the converted *M*_n_ values were calculated according to the Mark–Houwink relationship^54^ and the molar composition of the copolymer samples (determined by ^1^H NMR) as given in eqn (2) in the case of My/IBOMA copolymers:

2 *M*_n,MHS_ = *F*_My_[(*K*_P(MMA)_/*K*_P(My)_)*M*_n,GPC_^*α*_P(MMA)_+1^]^1/(*α*_P(My)_+1)^ + (1 − *F*_My_)[(*K*_P(MMA)_/*K*_P(IBOMA)_)*M*_n,GPC_^*α*_P(MMA)_+1^]^1/(α_P(IBOMA)_+1)^

Thermogravimetric analysis (TGA) was carried out using a Q500™ from TA Instruments under nitrogen flow at a ramp rate of 15 °C min^−1^. Samples were heated in aluminium pans. Differential scanning calorimetry (DSC, Q2000™ from TA Instruments) was used under N_2_ atmosphere. Indium was used as a standard to calibrate temperature while heat flow was calibrated *via* a benzoic acid standard. Three scans per cycle (heat/cool/heat) at a rate of 10 °C min^−1^ was set with a temperature range ranging for instance from −90 °C to +220 °C for the characterization of the triblock copolymers. Only the second heating run was considered to eliminate the thermal history. The reported *T*_g_s were calculated using the inflection method from the change in slope observed in the DSC traces.

The stress–strain features of the Co/IBOMA-My-IBOMA/Co triblock polymer (Co = S) were determined using a MTS Insight material testing system with a 5 kN load cell at room temperature and a cross-head speed of 10 mm min^−1^. Dog-bone style tensile specimens (ASTM D638 type V for reference, overall length = 63.5 mm, overall width = 9.53 mm) were prepared by solvent casting. To cast the film, the polymer sample was fully dissolved in dichloromethane (∼80 wt%) for an hour with continuous stirring using a magnetic stir bar. The solution was cast into a level Teflon Petri dish at room temperature for approximately two days. Then, the drying was completed after placing the film in the oven at 50 °C under vacuum until constant weight was measured. The film samples were cut into typical dog-bone shapes using a sharp blade. Specimens with defects (nicked sides, cracks, air bubbles) on their surface were discarded and were not used in mechanical testing. For each specimen, the thickness and the width were measured at five different points along the small centre portion with a digital caliper (Marathon, CO 030150F128) and the average film thickness and width were used for all calculations. The samples tested exhibited a mean thickness from 0.55 to 0.69 mm and a mean width from 3.11 to 3.32 mm. Each test was considered finished after the complete breakup of the specimen at the narrow section (films that broke near the grips were discarded). 6 specimens were tested, and averaged results were reported using TestWorks 4 software. The Young's modulus was determined as the slope of the stress–strain curve at strains of 0–0.5%.

The mechanical response of Co/IBOMA-My-IBOMA/Co (Co = S) to torsional oscillation at 0.15 Hz and 1% strain over a temperature range of 25 to 230 °C was measured at a rate of 5 °C min^−1^ under N_2_ using the CDT 450 convection heated measuring chamber mounted on the Anton Paar Modular Compact Rheometer MCR302. The solid rectangular fixture (SFR) was used, consisting of an upper and a lower holder with insets. Rectangular bars (thickness = 1.05 ± 0.04 mm; width = 8.91 ± 0.13 mm; length = 46.13 ± 0.24 mm) were made by solvent casting, following the above protocol used for preparing the tensile bars. Prior to the torsion tests, dynamic amplitude sweeps at 0.15 Hz and varying strain from 0.01 to 10% were conducted at various temperatures to ensure the material was kept within the linear viscoelastic regime (Fig. S12 in ESI†). Please note that, before the rheological characterization, the removal of the SG1 groups of Co/IBOMA-My-IBOMA/Co chain-ends was performed as described elsewhere.^44,55^

The micro-phase behaviour of Co/IBOMA-My-IBOMA/Co (Co = My) triblock polymer was studied by Atomic Force Microscopy (AFM). AFM images were collected in tapping mode using an AFM Bruker Multimode 8 equipped with Nanoscope V controller. The scanning speed for image acquisition was 0.5 Hz. The used cantilevers are Bruker TAP 300A with a radius of curvature of the tip of 8 nm. Data analyses were processed using the Nanoscope Analysis software (version 1.5). The samples were prepared as follows: the polymer (∼0.5 g) was fully dissolved in chloroform (∼10 mL) and one or two drops was spin coated on a silicon wafer, previously rinsed with acetone and cleaned by UV–O_3_ for 30 min. Lastly, the coated film was dried at 50 °C under vacuum overnight.

## Results and discussion

Two main parts are introduced thereafter and consist of the synthesis by nitroxide-mediated polymerization (NMP), *via* the SG1 nitroxide rate moderator, and the characterization of β-myrcene-rich (My-rich) and isobornyl methacrylate-rich (IBOMA-rich) copolymers. First, the nature of My/IBOMA copolymerization initiated by NHS-BlocBuilder (NHS-BB) was examined as well as its kinetics, its level of control and the ability of the resulting copolymers to re-initiate a second batch of monomer(s). Glass transition temperature (*T*_g_) and decomposition temperature (*T*_dec_) of the My/IBOMA copolymers were also investigated. Secondly, the preparation of relatively high *M*_n_ P(My)-(SG1)_2_ was explored by using the PEB-(SG1)_2_ dialkoxyamine. The possibility to synthesize triblock polymers by IBOMA/Co chain-extensions (Co = My or S co-monomer ∼ 8–9 mol%, relative to IBOMA) from P(My)-(SG1)_2_ was then presented. Scheme 1 depicts the various polymerizations done in this study.

### My/IBOMA copolymerization by NMP

#### Preliminary polymerization temperature study

Before exploring the effects of the initial mixture composition of the NMP-based My/IBOMA copolymerization over its kinetics and the composition of the resulting copolymers, the polymerization temperature was first studied. NMP of My in bulk initiated by NHS-BB at 120 °C was well-controlled, exhibiting minor deviations of *M*_n_ values from the predicted ones and low dispersities < 1.30, even at My conversion *X*_My_ > 50%.^29^ Interestingly, the copolymerization of My with methyl methacrylate (MMA, molar feed composition *f*_MMA,0_ = 0.50) and glycidyl methacrylate (GMA, *f*_GMA,0_ = 0.11–0.95) under the same experimental conditions also gave well-defined statistical copolymers.^56^ For both My/MMA and My/GMA systems, *M*_n_ increased linearly with overall conversion and *Đ* < 1.55 at the end of the reactions. Accordingly, these encouraging results prompted us to study two My/IBOMA copolymerizations in bulk using NHS-BB initiator at 120 °C (experiments My/IBOMA-50-T120 with *f*_My,0_ = 0.50 and My/IBOMA-70-T120 with *f*_My,0_ = 0.71, Table 1). Although a linear increase of *M*_n,MHS_ with the overall conversion *X* was observed in both cases, relatively broad molecular weight distributions were obtained throughout the reactions (*Đ* = 1.45–1.71 for *X* = 26–94%, Fig. S1, ESI†). This may be due to the likely high individual equilibrium constant of IBOMA, as typically reported for methacrylates,^25,57,58^ leading to a high concentration of propagating radicals that can promote self-termination. Consequently, it was decided to perform the two same experiments at 100 °C (experiments My/IBOMA-50 with *f*_My,0_ = 0.50 and My/IBOMA-70 with *f*_My,0_ = 0.70, Table 1) in order to know if a better control of the NMP can be obtained, by reducing likely the average activation–deactivation equilibrium constant 〈*K*〉 of this binary system. Regardless of *f*_My,0,_ similar *M*_n,MHS_ values were measured for the experiments run at 100 and 120 °C (Fig. S1, ESI†). However, narrower molecular weight distributions were obtained (*Đ* ≤ 1.29 for *X* = 9–65%) at 100 °C, for both experiments exhibiting *f*_My,0_ = 0.50 and 0.70. This apparent enhanced control of the NMP may be due to the reduction of disproportionation side reactions (β-hydrogen transfer from a propagating radical to SG1), observed during the NMP of methacrylates,^59,60^ at 100 °C. Accordingly, a copolymerization temperature of 100 °C was selected for the My/IBOMA NMP. This reduction of temperature is consistent with the SG1-mediated methacrylate polymerizations performed previously with a fairly low temperature (∼70–90 °C), notably by Charleux *et al.*^42,61^ and Lessard *et al.*^62,63^

#### Effect of feed composition on copolymer composition

It is first of interest to determine the monomer sequences of P(My-*co*-IBOMA) copolymers *via* the determination of the copolymer-monomer feed composition relationship. To that end, My/IBOMA NMP, with different feed ratios (*f*_My,0_ = 0.10–0.90), was carried out at 100 °C in bulk, initiated by NHS-BB and targeting *M*_n,theo_ = 30 kg mol^−1^, as indicated in Table 1. The copolymer composition was determined through ^1^H NMR analysis (*F*_My_ and *F*_IBOMA_ = 1 − *F*_My_, Fig. S5 in ESI† for the spectral assignments from experiment My/IBOMA-50) for relatively low overall conversion samples (*X* ≤ 24.5%, Table S1 in ESI† gives the samples used for extracting the reactivity ratios). The terminal copolymerization model, where only the terminal monomer residue is considered, was applied.^64^

**Fig. 1:**
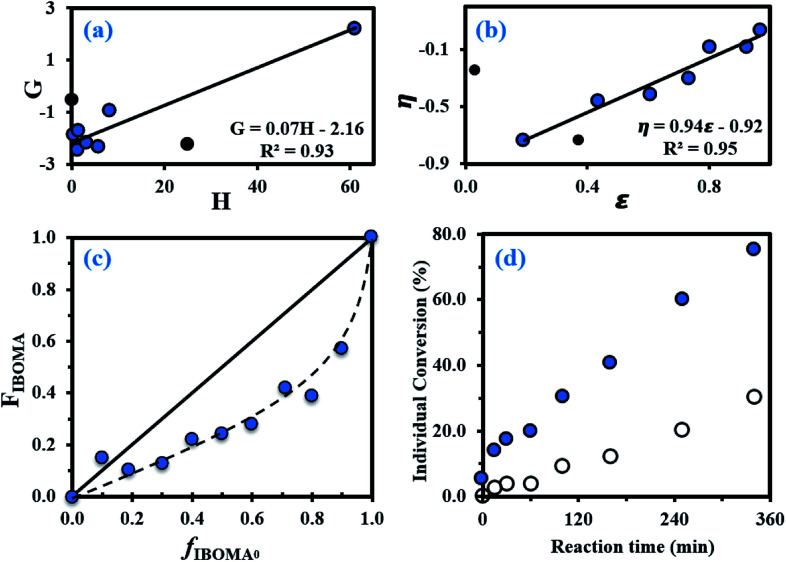
(a) Fineman–Ross (FR) and (b) Kelen–Tudos (KT) plots (solid black circles (●) corresponding to experimental outliers not taken into account for the calculations while solid lines refer to the linear trend lines) to determine the binary reactivity ratios for My and IBOMA for copolymerizations done in bulk at 100 °C initiated by NHS-BB (parameters *G*, *H*, *η* and *ε* defined in the ESI, page S4†). (c) Mayo–Lewis plot of My/IBOMA copolymerizations with respect to IBOMA, using the final molar composition *F*_IBOMA_, and the initial monomer feed composition *f*_IBOMA,0_. The solid straight line indicates the azeotropic composition where *f*_IBOMA,0_ = *F*_IBOMA_ while the dashed line is the associated trend line of the experimental data (solid blue circles (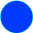)). Table S1 in the ESI† lists the samples used for these plots. (d) Individual My (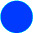) and IBOMA (○) conversions, determined by ^1^H NMR in CDCl_3_, *versus* reaction time *t* for the gradient copolymerization My/IBOMA-50 exhibiting *f*_My,0_ = 0.50.

The equations used for the calculation of the reactivity ratios can be found in the ESI (page S4).† By using the Fineman–Ross (FR) method (Fig. 1a),^65^ the reactivity ratios were determined: *r*_My_ = 2.16 ± 0.34 and *r*_IBOMA_ = 0.07 ± 0.04 (*r*_My_*r*_IBOMA_ = 0.15 ± 0.19). Similar values were calculated *via* the Kelen–Tudos (KT) approach (Fig. 1b):^66^*r*_My_ = 1.90 ± 0.18 and *r*_IBOMA_ = 0.02 ± 0.17 (*r*_My_*r*_IBOMA_ = 0.04 ± 0.13). The errors associated were derived from the standard errors of the slopes from FR and KT plots. Even though the KT equation refines the linearization method by introducing an arbitrary positive constant to spread the data more evenly, it remains a rearrangement of the copolymer composition equation and a non-linear least-squares (NLLS) fit to the Mayo–Lewis equation^67^ is likely the soundest method to determine *r*_My_ and *r*_IBOMA_. Consequently, the reactivity ratios determined by the KT method were used as the initial guesses for a NLLS fitting of the data (page S4 in ESI†). At 95% confidence level and with a regression coefficient *R*^2^ = 0.91, the statistical fit to the data yielded reactivity ratios *r*_My_ = 2.07 ± 0.58 and *r*_IBOMA_ = 0.05 ± 0.07. These latter results are similar to those obtained by FR and KT approaches, confirming the general trends with respect to My/IBOMA NMP at 100 °C in bulk and initiated by NHS-BB: *r*_My_ > 1.50, *r*_IBOMA_ < 0.30 and *r*_My_*r*_IBOMA_ < 0.50. The large difference in reactivity between My and IBOMA can be first noted. Whatever the terminal unit of the propagating species (⋯My˙ or ⋯IBOMA˙), the macro-radical showed a high preference for My. The insertion of IBOMA units at low conversions, either by self-propagation or cross-propagation, is not favoured: IBOMA-terminated macro-radical has a strong tendency to alternate. Accordingly, it can be assumed that My/IBOMA copolymerization under these conditions produces copolymers that possess a gradient in composition consisting of My enrichment at the start of the polymer chain followed by the incremental IBOMA enrichment at the end of the chain due to My depletion. However, the gradient nature of this copolymerization was moderate as indicated by the presence of methacrylate units at the start of the copolymer chain synthesized (*X* = 3–12% and *F*_IBOMA_ = 13–18% at *t* = 0–60 min for experiment My/IBOMA-50 for instance). Even though samples exhibiting relatively low conversions were used, it can be assumed that the composition drift in the monomer feed was non-negligible, especially taking the difference between *r*_My_ and *r*_IBOMA_ into account and was the main source of error by relying on the instantaneous copolymer composition model.^68^ To the best of our knowledge, no reactivity ratios were reported in the literature for diene/IBOMA systems, limiting thus the discussion. Nonetheless, for the My/GMA pair copolymerized under similar conditions (120 °C in bulk initiated by NHS-BB),^56^ statistical copolymers were synthesized with *r*_My_ = 0.48–0.80 and *r*_GMA_ = 0.50–0.71. Such a difference compared to the My/IBOMA system highlights the probable influence of the bulky and rigid bicyclic isobornyl groups over the reactivity of the methacrylate monomer (steric hindrance, hydrophobicity, electronic interactions/stabilization), as suggested by Trumbo as well.^69^

The Mayo–Lewis plot with respect to IBOMA is shown in Fig. 1c. Except for *f*_IBOMA,0_ = 0.10, the copolymer compositions are always below the diagonal line where the copolymer composition equals the feed composition (*F*_IBOMA_ = *f*_IBOMA,0_). Thus, the polymer formed at the early stages of the reaction was always richer in My than in IBOMA. The composition gradient of the My/IBOMA copolymers can be highlighted by determining the individual My and IBOMA conversions as a function of polymerization time, as illustrated in Fig. 1d with equimolar amounts of each monomer in the initial feed (*f*_My,0_ = 0.50). Throughout the first hour, essentially only My was reacting (*X*_My_ = 19.8% and *X*_IBOMA_ = 3.7% at *t* = 60 min) leading to essentially quasi P(My) propagating macro-radicals. Afterwards, while the consumption rate of My remained constant, IBOMA monomer reacted more significantly (*X*_IBOMA_ = 30.2% at *t* = 340 min) from a My-poor feed, resulting in an increase of the composition of IBOMA in the chain. Although this experiment was stopped at *X*_My_ = 75.2% and *X*_IBOMA_ = 30.2%, the last stages of the copolymerization can be easily deduced with the gradual IBOMA enrichment of the chain due to the depletion of My monomer.

#### Effect of feed composition on kinetics

Since the P(My-grad-IBOMA) gradient copolymers were characterized by GPC with THF at 40 °C and linear PMMA standards were used for the calibration, the method developed by Benoit *et al.* regarding the universal calibration^70^ was employed. As detailed in the Experimental section, Mark–Houwink–Sakurada (MHS) parameters for P(IBOMA) at 25 °C in THF eluent^52^ and for 1,4-P(My) at 30 °C in THF eluent^53^ were used and Fig. S2 (ESI)† shows *M*_n_ values before (*M*_n,GPC_) and after (*M*_n,MHS_) the MHS correction *versus* overall conversion *X* for experiments My/IBOMA-30 and My/IBOMA-80. Interestingly, while minor variations were observed for My-rich copolymers (experiment My/IBOMA-80 with *F*_My_ ≥ 0.66 during the whole reaction), as *F*_IBOMA_ increases, *M*_n,MHS_ becomes greater than *M*_n,GPC_ (My/IBOMA-30 with *F*_IBOMA_ ≥ 0.44 for *X* = 25–47%). It can be thereby hypothesized that an underestimation of IBOMA-rich copolymer *M*_n_ values was done when calibrating with PMMA due to the specific hydrodynamic volume of this copolymer coil in THF.

Before exploring the kinetics of My/IBOMA copolymerization at various initial feed compositions, it is useful to examine the homopolymerization of My and IBOMA by NMP under the same conditions (experiments My/IBOMA-100 and My/IBOMA-0, Table 1). My NMP at 100 °C in bulk mediated by NHS-BB showed a satisfactory control with *M*_n,MHS_ increasing linearly with conversion and *Đ* ≤ 1.36 (Fig. S4, ESI†), resulting in 1,4-rich P(My) (Table 2), even though non-negligible deviations of *M*_n,MHS_ values from theoretical ones were measured. The NMP of IBOMA without neither co-monomer nor additional free nitroxide was also attempted under these conditions. Controlled polymerization of methacrylates is still challenging for NMP because of their very high equilibrium constant *K* resulting from slow recombination of nitroxides and sterically-hindered poly(methacrylate) radicals.^71,72^ This long-time obstacle was confirmed once again by the inability to implement the SG1-based NMP of IBOMA in bulk at 100 °C in a controlled manner: the reaction medium polymerized spontaneously, giving P(IBOMA) chains exhibiting *M*_n,MHS_ = 27.1 kg mol^−1^ and *Đ* = 1.75 (My/IBOMA-0, Table 2). Due to the early high viscosity of the medium after only a few minutes, it was decided to repeat the experiment in 50 wt% toluene (experiment My/IBOMA-0-Tol, Table 1) in vain. The polymerization stagnated at *X* ∼ 10% with *M*_n,MHS_ = 11.4–12.3 kg mol^−1^ and *Đ* = 1.63–1.76 (Fig. S4, ESI†), illustrating presumably the disproportionation between P(IBOMA) macro-radicals and SG1 nitroxide.

**Fig. 2:**
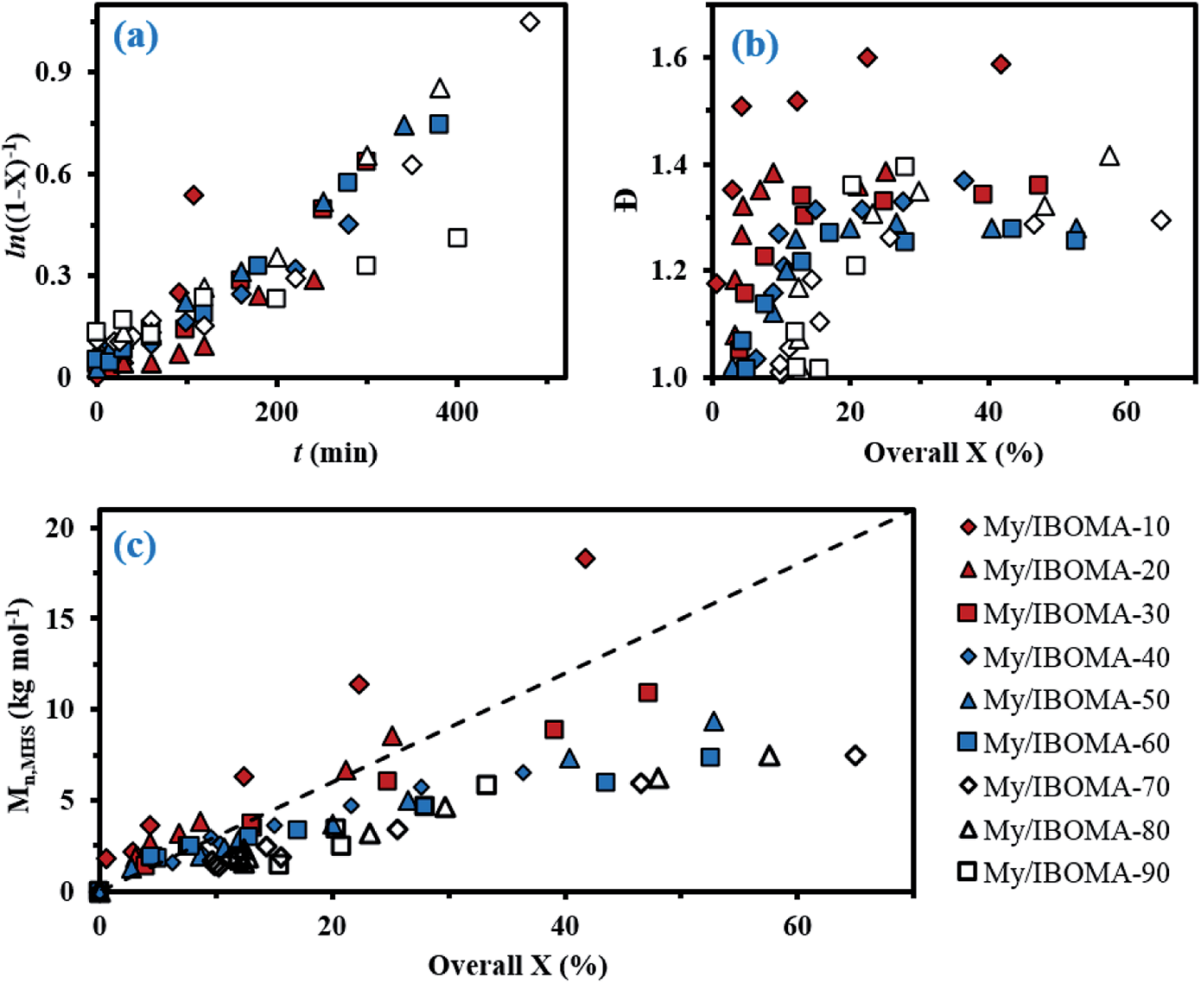
(a) Semi-logarithmic kinetic plots of ln((1 − *X*)^−1^) (*X* = overall conversion) *versus* polymerization time *t*, (b) *Đ versus* overall conversion *X* and (c) *M*_n_ determined by GPC relative to PMMA standards in THF at 40 °C, and corrected using the Mark–Houwink relationship, *versus* overall conversion *X* for the various My/IBOMA copolymerizations in bulk at 100 °C initiated by NHS-BB. The dashed line indicates the theoretical *M*_n_*versus* overall conversion based on the monomer to initiator ratio (*M*_n,theo_ ∼ 30 kg mol^−1^ at *X* = 100% for every experiment). All experimental ID and characterization of experiments are listed in Tables 1 and 2. The same legend at the bottom right of the figure is used for each of the three plots.

In order to produce IBOMA-rich polymers by SG1-mediated NMP, the controlling co-monomer approach, developed by Charleux and co-workers,^42–44^ was tried where the addition of a small concentration of a co-monomer exhibiting a much lower activation–deactivation equilibrium constant *K* with respect to the methacrylate enhances the control of the polymerization. It was herein decided to use My as a potential controlling co-monomer although its individual *K* has not been reported. 10–20 mol% of My had a marked impact on the outcome of My/GMA NMP initiated by NHS-BB in bulk. It gave a linear evolution of *M*_n_ with monomer conversion together with *Đ* ≤ 1.52 up to almost quantitative overall conversion.^56^ Previously, 10–20 mol% of isoprene, another conjugated 1,3-diene, allowed the well-controlled bulk NMP of *tert*-butyl acrylate and GMA.^19^ In the present study, the addition of 10 mol% of My (experiment My/IBOMA-10, Table 1) into the IBOMA feed undoubtedly slowed down the reaction even though the features of a controlled system were only partially observed due to the upward curvature of the semi-logarithmic plot and *Đ* = 1.51–1.60 at *X* = 4–42% (Fig. 2a and b). Expectations were fully met by performing the NMP of IBOMA in the presence of 20 mol% of My as a co-monomer (My/IBOMA-20, Table 1) with *Đ* ≤ 1.39, *M*_n_ increasing linearly with *X* and a quasi-linearity of ln((1 − *X*)^−1^) *versus t* during the reaction with *R*^2^ = 0.92 (Fig. 2a–c). It can be assumed that the addition of My in the IBOMA-rich feed induced a significant decrease of the average 〈*K*〉 and therefore enhanced the control of the polymerization.

**Fig. 3:**
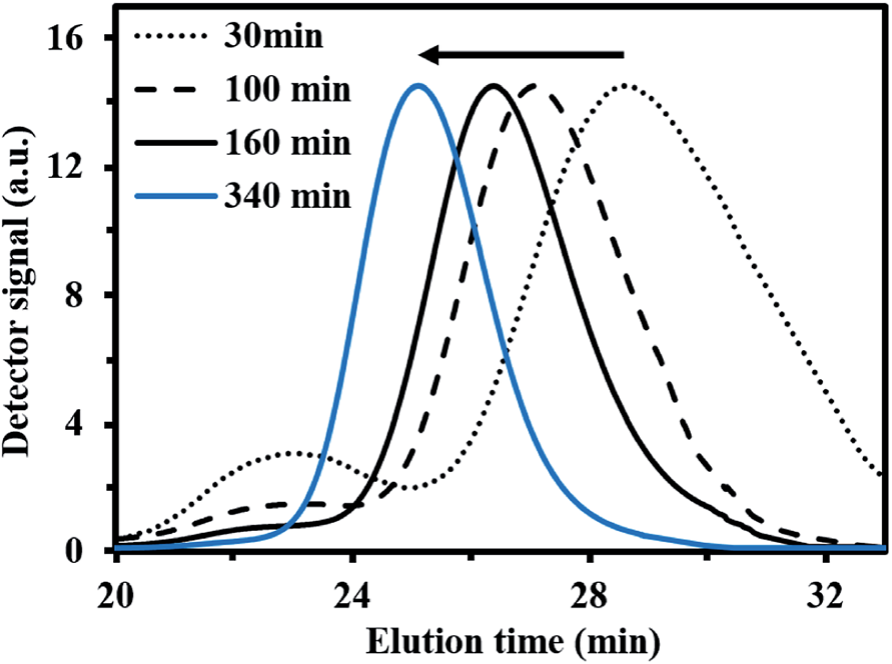
Normalized GPC traces of P(My-grad-IBOMA) with *f*_My,0_ = 0.50, initiated by NHS-BB at 100 °C in bulk targeting *M*_n,theo_ = 30 kg mol^−1^ at *X* = 100% (experiment My/IBOMA-50).

For *f*_My,0_ = 0.30–0.90, linear *M*_n,MHS_ increase with overall *X* and narrow molecular weight distributions (*Đ* ≤ 1.41) of the P(My-grad-IBOMA)s were observed (Fig. 2b and c). These results presumably illustrate the effective mediation of the stable SG1 free nitroxide, capping the propagating chains and thus shifting the equilibrium toward deactivation.^12^ Although termination and transfer side reactions were still present, they were likely minimized due to the high number of reversibly terminated chains (dormant P(My-grad-IBOMA)-SG1 chains). Nonetheless, as *f*_My,0_ increased, the deviations of *M*_n,MHS_ from *M*_n,theo_ became more significant. Even though the principle of universal calibration was applied, the MHS coefficients did not match exactly the GPC conditions used (THF, 40 °C). The likely large differences in hydrodynamic volumes of the P(My-grad-IBOMA)s and that of the PMMA standards that was used to calibrate the GPC may partly explain the deviations. The copolymerization with *f*_My,0_ = 59–90% had a flatter slope compared to the others (Fig. 2c), where the unreliability of the GPC calibration cannot be the sole reason. Chain transfer side reactions to the monomers, creating new chains and resulting in shorter average chain length, may also explain the loss of control.^73^ The GPC chromatograms at different polymerization times are shown in Fig. 3 for the experiment My/IBOMA-50. The monomodal shift of the curves to lower elution times with no major tails confirmed the well-controlled NMP of this monomer pair. As reported previously with regard to the homopolymerization of My initiated by NHS-BB in bulk at 120 °C,^29^ a minor peak, corresponding to high *M*_n_ polymers, was detectable mostly at the commencement of the copolymerization (20–25 min, Fig. 3), presumably due to My auto-initiation. Indeed, the spontaneously free radical polymerization of My was reported at elevated temperatures as well as at room temperature.^74^ Under this assumption, My auto-initiation during the NMP of the My/IBOMA mixture would result in the presence of extra active species, not necessarily mediated by the nitroxide radical.

Attention can also be paid to the kinetic plots of ln((1 − *X*)^−1^) *versus* reaction time *t* (Fig. 2a). For *f*_My,0_ = 0.10–0.81, all copolymerizations obeyed first order kinetics as described by eqn (3) and generated good fits to linear kinetic plots (squared linear regression coefficient *R*^2^ ≥ 0.94 for the whole experiments).

3 ln([M]_0_/[M]_*t*_) = ln([M]_0_/([M]_0_(1 − *X*))) = ln((1 − *X*)^−1^) = 〈*k*_p_〉[P˙] time

In eqn (3), [M]_0_ and [M]_*t*_ are the concentrations of monomer at time zero and subsequent later time *t*, respectively, 〈*k*_p_〉 is the average propagation rate constant and [P˙] is the propagating radical concentration. Marked upward curvatures (Fig. 2a) can be seen for experiments My/IBOMA-10 and My/IBOMA-20 having an initial feed rich in IBOMA (*f*_IBOMA,0_ = 0.80–0.90). It suggests an increase in [P˙], which can be explained by the contribution of the likely high individual *K* of IBOMA, resulting from slow recombination of nitroxides and poly(methacrylate) radicals (decrease in the concentration of dormant chains) and leading to a high [P˙]. 〈*k*_p_〉[P˙] values were determined from the slopes of the semi-logarithmic plots of conversion *versus* time (Fig. 2a) in the linear regions where [P˙] is expected to remain relatively constant (see Table 2, footnote *d*). These slopes can be related to the equilibrium between the dormant and the active chains as shown in eqn (4).

4 *K* = ([P˙][SG1˙])/[P-SG1]

where [SG1˙] and [P-SG1] are the concentrations of free nitroxide and dormant alkoxyamine terminated polymer, respectively. At the early stages of the copolymerization, it can be hypothesized that [P-SG1] = [NHS-BB]_0_ (one initiating moiety generates a dormant chain) as well as [SG1˙]_0_ = [SG1˙] (high initial amount of free nitroxide being relatively constant in the course of the reaction). Eqn (4) can therefore be rewritten in terms of *r* = [SG1]_0_/[NHS-BB]_0_.

5 〈*k*_p_〉〈*K*〉 ≈ (〈*k*_p_〉[P˙][SG1]_0_)/[NHS-BB]_0_ = 〈*k*_p_〉[P˙]*r*

Initially, we expected significant differences in 〈*k*_p_〉〈*K*〉 values between My-rich and IBOMA-rich initial feeds. Indeed, even though no kinetic constants for My are reported yet, *k*_p,I_ = 125 ± 30 L mol^−1^ s^−1^ for the radical polymerization of isoprene, another similar conjugated 1,3-diene, initiated by di-*tert*-butyl peroxide at 5 °C, was reported.^75^ Comparatively, *k*_p,IBOMA_ ∼ 280 L mol^−1^ s^−1^ at 5 °C can be estimated from the study led by Beuermann and co-workers using the pulsed-laser polymerization-GPC technique.^76^ IBOMA and My homopolymerizations performed herein (My/IBOMA-0 and My/IBOMA-100, Table 1) confirmed the kinetic singularity of these two monomers polymerized by NMP. While a quasi-spontaneous polymerization was performed when an IBOMA solution was heated at 100 °C in the presence of NHS-BB, My polymerized slowly under identical experimental conditions and this reaction exhibited *k*_p_*K* = (1.4 ± 0.4)10^−5^ s^−1^ (Table 2) consistent with the kinetic values reported for the NMP of dienes.^27,29^ Very interestingly, 〈*k*_p_〉〈*K*〉 was on average (2.03 ± 0.94)10^−5^ s^−1^ for the NMP of My with IBOMA with *f*_IBOMA,0_ = 0.10–0.90 (Table 2), which was substantially close to the 〈*k*_p_〉〈*K*〉 value of the NMP of My. Accordingly, it can be assumed that the copolymerization kinetics was largely governed by the My kinetics, even when the initial mixture was rich in IBOMA. This behaviour is illustrated in Fig. 2a where all curves almost overlap, regardless of *f*_IBOMA,0_.

The molar fractions of 1,4-, 1,2- and 3,4-P(My) additions in the My/IBOMA copolymers were determined by ^1^H NMR (Fig. S5 in ESI† for the assignments). Interestingly, while the experiment My/IBOMA-100 led to a P(My) homopolymer rich in 1,4-content (86.6 mol%, Table 2), the fraction of 1,2-motif in the copolymers markedly increased when IBOMA-rich feeds were used (∼30 mol% of 1,2-content for *f*_IBOMA,0_ = 0.41–0.80). A very similar trend was observed for the systems My/GMA and My/*tert*-butyl acrylate copolymerized under the same conditions at 120 °C and 115 °C respectively.^56^ Therefore, it must be caused by the presence of a methacrylate radical as a terminal unit, which might have altered the steric and/or the electronic environment of the active end of the propagating species.

#### Glass transition and decomposition temperatures of P(My-grad-IBOMA)

Differential scanning calorimetry (DSC, see Experimental section) was used to evaluate the nature and breadth of the gradient copolymer P(My-grad-IBOMA) glass transitions. Attention was first paid to the glass transition temperatures (*T*_g_s) of the respective homopolymers, P(My) and P(IBOMA). The *T*_g_ of NMP-based P(My) was previously estimated at −77.0 °C,^29^ highlighting its elastomeric nature. *T*_g,P(IBOMA)_ = 171–177 °C was measured *via* the DSC curves of dry P(IBOMA)s from experiments My/IBOMA-0 and My/IBOMA-0-Tol, performed in bulk and in 50 wt% toluene, respectively. This elevated *T*_g_ is explained by the limited P(IBOMA) segment flexibility caused by the steric hindrance of the bulky isobornyl group. Relying on previous studies,^40,41,77^ the relatively low *T*_g_s observed herein for these rigid blocks suggest that P(IBOMA)s rich in isotactic triads (>50 mol%) were produced. Indeed, Yu *et al.* notably showed that syndiotactic P(IBOMA) (>50 mol%) exhibited a higher *T*_g_ = 195–206 °C.^41^

**Fig. 4:**
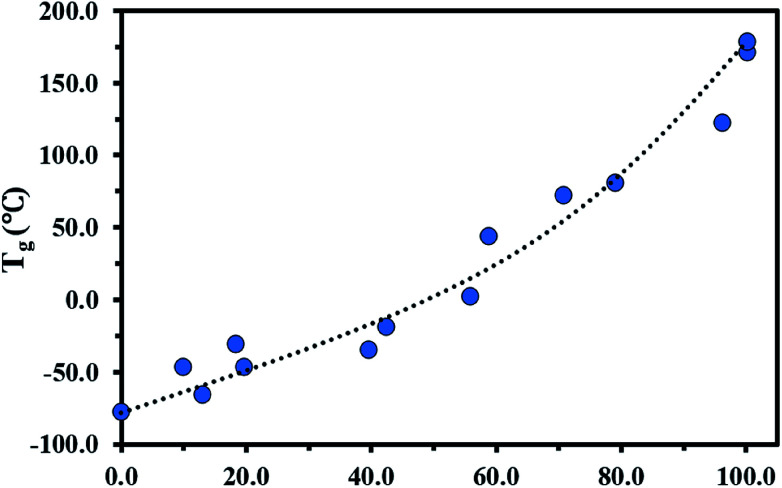
*F*_IBOMA_ effects on *T*_g_ in P(My-grad-IBOMA) gradient copolymers. The DSC traces can be found in the ESI, Fig. S7.†*T*_g_ = −77.0 °C for *F*_IBOMA_ = 0 was determined previously.^29^ The black dotted line represents the experimental data fitted to the Gordon–Taylor equation.

The glass transition behaviour of P(My-grad-IBOMA) copolymer with cumulative IBOMA mole fractions ranging from 0.10 to 0.96 was also characterized using DSC heat curves (Fig. S7, ESI†). *T*_g_*versus* IBOMA composition plot is given in Fig. 4. A trend curve was included, which was obtained by fitting the Gordon–Taylor equation^78^ (eqn (6)) to the experimental data.

6 *T*_g_ = (*T*_g,P(My)_*w*_My_ + *KT*_g,P(IBOMA)_*w*_IBOMA_)/(*w*_My_ + *Kw*_IBOMA_)

where *w*_My_ and *w*_IBOMA_ are the weight fractions and *T*_g,P(My)_ and *T*_g,P(IBOMA)_ the glass transition temperatures of the respective homopolymers. *T*_g_ corresponds to the glass transition temperature of the gradient copolymer and *K* = Δ*β*_IBOMA_/Δ*β*_My_ with Δ*β*_IBOMA_ or Δ*β*_My_ being the difference between the expansion coefficients of the rubbery and glassy states of P(IBOMA) or P(My). This theory is based on two basic assumptions: the volume additivity (ideal volume of mixing) and a linear change in volume with temperature. With 95% confidence bounds, *K* = 0.322 ± 0.090 (*R*^2^ = 0.94) was determined *via* the use of a non-linear least-squares fitting of the data and indicated the larger expansivity of My units in the rubbery state than that of IBOMA units. A similar trend was reported for the system My/GMA (*K* = 0.186 ± 0.036),^56^ highlighting the plasticizing nature of P(My). The Gordon–Taylor fitting approach yielded a good representation of the experimental *T*_g_ data (Fig. 4). A slight concave *T*_g_*versus* IBOMA composition curve was obtained, pointing out deviations from an ideal mixing. As expected, P(My-grad-IBOMA)'s *T*_g_ increased from −65 to 123 °C with *F*_IBOMA_ ranging from 9.8 to 96.2 mol%. Interestingly, the presence of a minor fraction of My units considerably reduced the *T*_g_ of the copolymer (*T*_g_ = 81 °C for *F*_My_ = 0.21 in comparison to *T*_g,P(IBOMA)_ ∼ 174 °C) while a moderate increase of the *T*_g_ was observed for a My-rich P(My-grad-IBOMA) containing a minority of IBOMA units (*T*_g_ = - 46 °C for *F*_IBOMA_ = 0.20 and *T*_g,P(My)_ = - 77 °C). Two explanations can be suggested: the long C6/C8 pendant groups of My units generating significant free volume; the gradient nature of the copolymer with a largely higher reactivity for My, resulting in the formation of short P(My) segments (elastomeric “knots”) and the isolation of the isobornyl rings.

**Fig. 5:**
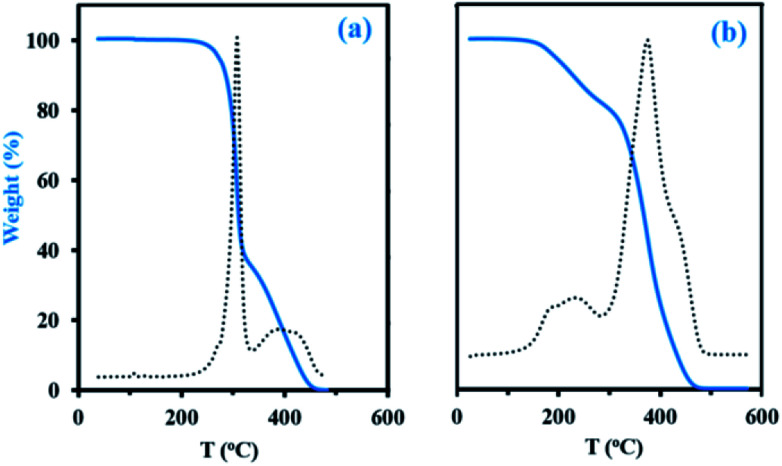
TGA traces (N_2_ atmosphere, 10 °C min^−1^) of final gradient copolymers (a) My/IBOMA-20 and (b) My/IBOMA-80, previously precipitated in excess methanol and dried under vacuum at 50 °C. Sample weight *versus* temperature is represented by the solid blue line whereas the dotted line represents the derivative of weight relative to the temperature *versus* temperature in order to determine precisely the temperature at which weight loss is most apparent (*T*_dec,max_).

Thermogravimetric analysis (TGA, Experimental section) has been applied to decomposition study on the final dry samples taken from experiments My/IBOMA-20 and My/IBOMA-80, exhibiting 51 and 81 mol% of My units respectively (Table 2). Fig. 5 represents the thermal decomposition curve of both gradient copolymers. Interestingly, the synthesized polymers exhibited markedly a two-step degradation pattern. A first weight loss was observed from 145 to 305 °C with a *T*_dec,max_ of 242 °C (temperature at which the weight loss is most apparent, maximum of the derivative curve) for the sample richer in My (My/IBOMA-80, Fig. 5b) and from 205 to 315 °C with *T*_dec,max_ = 307 °C for My/IBOMA-20 sample (Fig. 5a). This early degradation peak was likely due to the loss of the side chains by cleavage of the *O*-isobornyl bond, releasing camphene as a main product, as reported by Matsumoto and coworkers.^40^ This first weight loss was proportional to the IBOMA fraction possessed by the samples: while about 40% of mass loss was measured for My/IBOMA-20 with *F*_IBOMA_ = 0.49, only about 22 wt% of My/IBOMA-80, exhibiting *F*_IBOMA_ = 0.19, was lost (Fig. 5). This observation supports the contribution of the IBOMA units to the premature P(My-grad-IBOMA) thermal decomposition. The second degradation step is similar for both samples, starting at 305–315 °C with *T*_dec,max_ = 370–380 °C and decomposing totally the copolymers at about 450 °C (Fig. 5), which could be mostly caused by the scission reactions of the main chain (depolymerization) resulting in the formation of monomer and oligomer radicals. We previously reported the decomposition behaviour of SG1-mediated P(My), from 290 to 485 °C (*T*_dec,max_ = 385 °C),^29^ matching closely the second degradation step of the two P(My-grad-IBOMA)s characterized.

#### Chain-end activity of NMP-based P(My-grad-IBOMA)

Ideally, a majority of the P(My-grad-IBOMA) chain ends synthesized by NMP should possess the SG1 moiety. Indeed, the recombination of the propagating macro-radical with the controlling nitroxide should lead to a very low [P˙], limiting the irreversible self-termination reaction. Afterwards, the final SG1-terminated P(My-grad-IBOMA) should be re-activated *via* the thermal homolytic decomposition of this dormant species into the initiating macro-radical and the SG1 persistent radical. Two strategies were herein adopted to know if P(My-grad-IBOMA) chains were effectively capped by a SG1 group: ^31^P NMR analysis (see Experimental section) allowing the detection of the phosphorus atom of the nitroxide group;^79,80^ IBOMA and/or My chain-extension from P(My-grad-IBOMA) macro-initiator by the same nitroxide mediated mechanism. Two samples were studied: My/IBOMA-82 and My/IBOMA-44 exhibiting respectively 82 and 44 mol% of My (Table 3A).

^31^P NMR was first performed to evaluate the living fraction (LF) of these two gradient copolymers initiated by the NHS-BB alkoxyamine (Fig. S6 in ESI for the spectra). The proportion of chains containing a SG1 end group was 74 ± 9 mol% and 69 ± 3 mol% for My/IBOMA-44 and My/IBOMA-82 copolymers, respectively (Table 3A). It is interesting to note that about two thirds of the My-rich My/IBOMA-82 chains remained SG1-capped despite a relatively high overall conversion (*X*_1_ = 64.2%). It can thus be deduced that about 30% of these chains underwent irreversible terminations, becoming more prevalent at high conversion, resulting notably in a relatively broad molecular weight distribution (*Đ*_1_ = 1.51, Table 3A). A similar LF was measured for the IBOMA-rich gradient copolymer exhibiting a lower overall conversion (*X*_1_ = 39.1%). A moderate conversion was targeted for the synthesis of this macro-initiator since an IBOMA-rich (*f*_IBOMA,0_ = 0.70) feed was polymerized, where disproportionation side reactions likely occur. It can be assumed that the synthesis of a high conversion P(My-grad-IBOMA) rich in IBOMA would have a relatively low LF, caused by the occurrence of terminations by H-transfer notably.

**Fig. 6:**
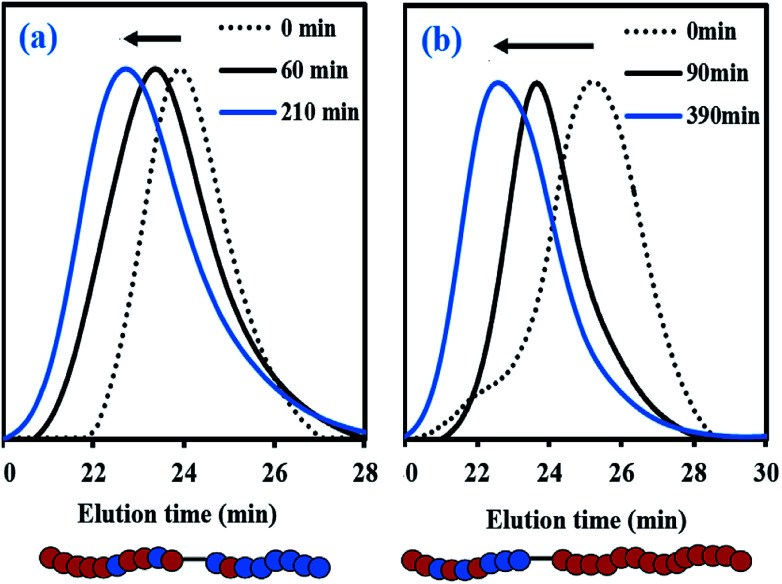
Normalized GPC traces for the chain-extensions of (a) My/IBOMA-82 with a IBOMA/My (93/7 mol%) mixture (experiment My/IBOMA-82-IBOMA/My) and (b) My/IBOMA-44 with My (experiment My/IBOMA-44-My) at *T* = 105–115 °C in 50 wt% toluene. Schematic representations of the final chain-extended copolymers, where solid red and blue circles represent My and IBOMA units respectively, are given below the GPC chromatograms.

To further examine the capacity of these samples to re-initiate and produce diblock copolymers, two chain-extension experiments were done using My/IBOMA-44 and My/IBOMA-82 as macroinitiators (Table 3A). The reaction conditions and results of the chain-extensions are given in Tables 3B and C respectively. My/IBOMA-44 macroinitiator (*F*_IBOMA,1_ = 0.56, *M*_n,MHS,1_ = 8.8 kg mol^−1^) was cleanly chain-extended at 115 °C in toluene with My to obtain a P[(My-grad-IBOMA)-*b*-My] diblock copolymer (*F*_IBOMA,2_ = 0.07, *M*_n,MHS,2_ = 30.1 kg mol^−1^, Table 3C) containing predominantly My units. The dispersity of this chain-extended product (*Đ*_2_ = 1.65) was higher than that of the parent macro-initiator (*Đ*_1_ = 1.32), which suggests that termination occurred during the reaction and/or some macroinitiator was not initiated. This latter assumption can be supported by the previous quantitative spectroscopic analysis, indicating that about 25 mol% of My/IBOMA-44 was not terminated by SG1 (Table 3A), as well as the GPC traces of the chain-extension experiment (Fig. 6b) showing that the tails of the final chain-extended copolymer overlapped to some degree with the associated macroinitiator trace. The asymmetrical final GPC trace at 390 min may also indicate the occurrence of irreversible terminations throughout the chain-extension, which generate “dead” species having lower *M*_n_ (∼15–25 kg mol^−1^). Generally, this successful My chain-extension from IBOMA-rich P(My-grad-IBOMA) copolymer prompted us to investigate the reversed situation where a My-rich macroinitiator can be re-activated to add an IBOMA-rich segment. With a fresh IBOMA/My mixture (93 mol% of purified IBOMA), My/IBOMA-82 (*F*_My,1_ = 0.82, *M*_n,MHS,1_ = 13.6 kg mol^−1^, *Đ*_1_ = 1.51, Table 3A) was extended at 105 °C in 50 wt% toluene (Table 3B). Due to the beneficial kinetic effects observed of the addition of 10–20 mol% of My over the control of the SG1-based NMP of IBOMA (Fig. 2), 7 mol% of the acyclic monoterpene was initially introduced in the feed. After 210 min, the resulting chain-extended diblock exhibited *M*_n,MHS,2_ = 23.2 kg mol^−1^, *Đ*_2_ = 1.61 and the overall My molar composition was decreased from 82 to 49 mol% (Table 3C). Similar to the first chain-extension, the GPC trace of the chain-extended product retained generally its mono-modal nature and had a clear shift to the left (Fig. 6a), indicating a high-level of chain-end fidelity of the original macroinitiator.

These satisfactory results naturally paved the way to the preparation by NMP of well-defined 1,3-diene-based triblock copolymers composed of a flexible and bio-sourced P(My) mid-segment^33^ and two rigid P(IBOMA) outer segments, the latter coming from partially bio-based raw material source.^81,82^

### Co/IBOMA-My-IBOMA/Co triblock copolymers by NMP

The synthesis by SG1-mediated NMP of triblock copolymers consisting of a soft P(My) middle block and two hard blocks, containing mostly IBOMA units, was aimed in this second part. A two-step approach was applied for preparing these triblock copolymers: (1) NMP of My initiated by the PEB-(SG1)_2_ difunctional initiator (telechelic poly(ethylene-*stat*-butylene) copolymer terminated with SG1 groups);^49^ (2) chain-extension of the P(My)-(SG1)_2_ macroinitiator with a IBOMA/Co mixture (Co = My or S co-monomer, ∼8–9 mol% relative to IBOMA). The synthesis route is depicted in Scheme 1b. A triblock molecular architecture was targeted so that both ends of the rubbery mid-segment are theoretically anchored to a glassy domain,^83^ mimicking thus the traditional micro-structure of styrene block copolymers such as SIS or SBS (I = isoprene, B = butadiene).

#### Optimized synthesis of P(My)-(SG1)_2_

First, the preparation of a P(My) segment exhibiting a relatively high *M*_n_ and able subsequently to re-initiate a fresh batch of monomer(s) at each chain end was desired. In order to have sufficient mechanical properties, the targeted final triblock must have an entangled elastomeric phase, and the physical chain entanglements must be contributing mainly to the network density and to the stress at small elongations.^84^ P(My) entanglement molecular weight (*M*_e,P(My)_ = 22–31 kg mol^−1^) is significantly higher than that of comparable poly(diene)s such as poly(isoprene) PI (*M*_e,PI_ = 4–6 kg mol^−1^) or poly(butadiene) PB (*M*_e,PB_ = 2–4 kg mol^−1^).^85^ Accordingly, *M*_n_ > 40 kg mol^−1^ for P(My)-(SG1)_2_ was desired to ensure a minimal entanglement density.

PEB-(SG1)_2_ dialkoxyamine macroinitiator (*M*_n,MHS_ = 5.7 kg mol^−1^, *Đ* = 1.17) was first produced, according to the method reported by Lessard *et al.*^49^^1^H NMR of the final product revealed that about 84 mol% of the chain ends were capped by a SG1 unit. It can thus be deduced that a minor fraction of the synthesized macroinitiator consisted of monofunctional alkoxyamine terminated by SG1 only at one chain end and “dead” PEB chains. To know the optimal conditions for making high average chain length P(My) in a controlled manner, NMP of My initiated by various concentrations of PEB-(SG1)_2_ at 120 °C in bulk was then pursued. Four different theoretical *M*_n_ were thus targeted at quantitative conversion, namely 79.1, 105.0, 137.2 and 174.0 kg mol^−1^ (Table S2, ESI†), and kinetic studies were led (Fig. S8, ESI†) to assess the level of control. As *M*_n,theo_ increased, higher dispersity values and greater deviations of *M*_n,MHS_ from the theoretical molar masses were observed (Fig. S7b and S7c†), confirming likely the greater presence of irreversible terminations and chain transfer reactions to My and P(My) with [My]_0_. Nonetheless, lowering the dialkoxyamine concentration resulted in the synthesis of higher *M*_n,MHS_ P(My)s. Particularly, when targeting *M*_n,theo_ = 137.2 kg mol^−1^ at *X*_My_ = 100% (experiment My-137), a polyterpene exhibiting *M*_n,MHS_ = 49.5 kg mol^−1^ and *Đ* = 1.52 was obtained at 51% conversion (Fig. S7b and S7c†), which consisted of the most satisfactory balance between a relatively high *M*_n_ and a moderate dispersity. It was then attempted to produce longer P(My) chains with a relatively narrow molecular weight distribution by using a low concentration of the dialkoxyamine initiator [PEB-(SG1)_2_] (*M*_n,theo_ = 169–173 kg mol^−1^ at *X* = 1.0) combined with additional free SG1 nitroxide (9–18 mol% with respect to the initiator, experiments My-173_SG1,9_ and My-169_SG1,18_, Table S2†). An improved control of the NMP when using a slight excess of this potent nitroxide could have been expected since the dormant state should be favoured (lower concentration of propagating macro-radicals, reduction of the overall polymerization rate).^86–88^ Despite apparent rate constants somewhat lower as [SG1˙]_0_ increased (Fig. S9a in ESI†), similar *Đ versus X* and *M*_n,MHS_*versus X* trends were determined (Fig. S8b and S8c†) indicating the ineffectiveness of adding SG1 free radical to the reaction mixture. This observation echoes a previous study showing that the addition of 4.9–11.7 mol% of the SG1 mediator with respect to the initiator did not enhance the NMP of My at 120 °C in bulk initiated by NHS-BB.^29^ A last experiment in 50 wt% toluene, without extra SG1, was led, predicting *M*_n,theo_ ∼ 170 kg mol^−1^ again (experiment My-170_Tol_, Table S2†). The use of a solvent was thought to help, as seen by the increase in viscosity at *X*_My_ > 30%, when performing this reaction in bulk, which might account for the occurrence of diffusional limitations,^89^ bringing about a loss of control. Interestingly, while the polymerization in toluene was slower (Fig. S8a†), similar molecular characteristics of the growing P(My) were obtained compared to the previous mass polymerizations (Fig. S8b and S8c†). Higher *Đ* values were even observed (Fig. S8b†), which may be assigned to chain-transfer side reactions to toluene.^90,91^

Consequently, the P(My) segment was prepared *via* the PEB-(SG1)_2_-initiated NMP of My at 120 °C in bulk for 280–400 min (*X*_My_ ∼ 40–50%) targeting *M*_n,theo_ ∼ 140 kg mol^−1^ and without any additional free nitroxide. Two 1,4-rich P(My)s (experiments My-35 and My-52, Table 4A), exhibiting *M*_n,MHS_ = 35–52 kg mol^−1^ and acceptable *Đ* = 1.45–1.53, were synthesized this way. Attention was now shifted to the capacity of these P(My) segments, ideally capped by two SG1 units, to form triblock copolymer with a mixture of IBOMA containing a minor fraction of co-monomer.

#### IBOMA/Co chain-extension from P(My)-(SG1)_2_

IBOMA/My chain-extension (7.8 mol% of My used as a co-monomer) from My-35 macroinitiator was first performed at 115 °C in toluene for 90 min (Table 4B). The shifts in the GPC traces, seen in Fig. S10a (ESI),† clearly show the shift in elution time demonstrating the increase in *M*_n_ with time. The chain-extended product My-35-IBOMA/My had an increase in *M*_n_ from 35.2 to 51.3 kg mol^−1^ with a broadening of the molecular weight distribution going from 1.45 to 1.91. The monomodal nature of the GPC chromatograms confirmed that the majority of the My-35 chain ends were reversibly terminated. Based on ^1^H NMR analysis, the final triblock copolymer exhibited an overall *F*_IBOMA_ = 0.26 (Table 4C). More specifically, each chain-extended block was composed of a high fraction of IBOMA units (91 mol%) and a minority of My (9 mol%).

Afterwards, a second triblock copolymer, with a higher overall *M*_n_ as well as harder outer blocks, was targeted. To that end, My-52 macroinitiator (*M*_n,MHS,My_ = 51.7 kg mol^−1^, *Đ* = 1.53, Table 4A) was reacted with a IBOMA/S mixture (8.7 mol% of S used as a controlling co-monomer, as previously reported^24,42,92^) for 3 h in 50 wt% toluene at 115 °C (Table 4B). The resulting product My-52-IBOMA/S had *M*_n,MHS,CE_ = 78.1 kg mol^−1^ and *Đ* = 2.41. Likewise, the final chain-extended product shifted to lower elution time compared to the macroinitiator (Fig. S9b†). To increase My-52-IBOMA/S *M*_n_, a fractional precipitation using the non-solvent addition method was implemented, relying on the benzene (solvent)/methanol (non-solvent) pair. Eventually, the fractionated triblock copolymer had *M*_n,MHS_ = 94.7 kg mol^−1^ and *Đ* = 2.23 (Table 4C), reflecting the removal of a fraction of relatively short chains. The rigid segments were constituted of about 88 mol% of methacrylate units and 12 mol% of S, as determined by ^1^H NMR.

A high *Đ* = 1.91–2.23 was measured for these final triblock copolymers, which may be mainly caused by the difficulty to control the polymerization of a methacrylic ester-rich feed (*f*_IBOMA,0_ ≥ 0.91), although a small amount of My or S were added to possibly decrease the average activation–deactivation equilibrium constant 〈*K*〉. The inactivity of a fraction of PEB-(SG1)_2_ and P(My)-(SG1)_2_ macroinitiators may also contribute to the broadening of the molecular weight distribution. Besides, the very likely presence of PEB-SG1 monofunctional chains may have resulted in the synthesis of short diblock copolymer chains, assuming a similar activity between PEB-SG1 and PEB-(SG1)_2_. All these hypotheses would lead to a non-negligible population of shorter than expected chains, as observed in Fig. S10 (ESI†) by the tails on the low molecular weight edge of the GPC chromatograms (elution time ∼ 23–25 min).

#### Characterization of the NMP-based Co/IBOMA-My-IBOMA/Co triblock copolymers

**Fig. 7:**
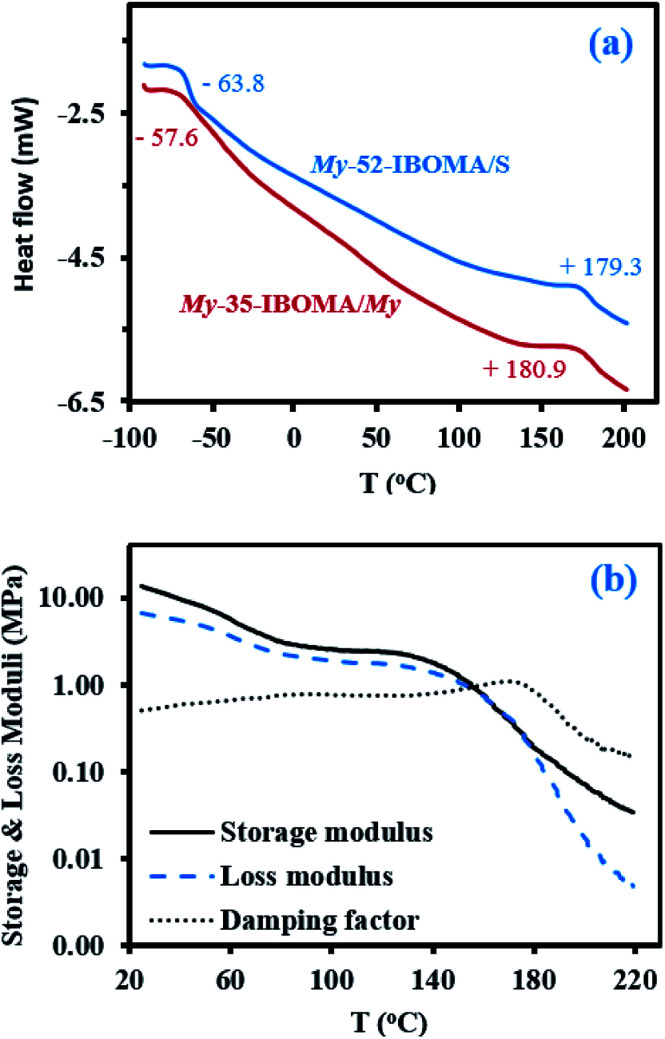
(a) DSC traces (second heating run) of the triblock copolymers My-52-IBOMA/S (blue) and My-35-IBOMA/My (red). The numbers near the changes in slope correspond to the *T*_g_s determined *via* the inflection method. (b) Dynamic mechanical analysis of the sample My-52-IBOMA/S by torsional oscillation, yielding the storage modulus, the loss modulus and the damping factor *versus* temperature (0.15 Hz, 1% strain, 5 °C min^−1^, N_2_ atmosphere).

The Co/IBOMA-My-IBOMA/Co triblock copolymers investigated were characterized by two discrete transition regions indicative of each polymer segment type (Fig. 7a). A lower *T*_g_ of about −60 °C corresponded to the transition of the P(My) mid-segment while a largely upper transition at around +180 °C was assumed to indicate the softening of the IBOMA-rich segments. The lower *T*_g_ of these triblock polymers is about 15 °C higher than that of 1,4-rich P(My) homopolymer (*T*_g,P(My)_ = −77 °C, Fig. 4), which might be due to some partial compatibility of P(My) and IBOMA-rich regions. It is of interest to note that the upper *T*_g_ of the synthesized triblocks is similar to that of P(IBOMA) homopolymer (*T*_g,P(IBOMA)_ = 171–177 °C, Fig. 4) even though the outer hard blocks contained a non-negligible fraction of S (12 mol%) or My (9 mol%) units. A decrease of the upper *T*_g_ would have been expected due to the presence of softer units in the rigid segments (IBOMA/My system illustrated in Fig. 4). This unaffected upper *T*_g_ can result from the extended blocks composition. For the IBOMA/My chain-extension, it can be assumed that the majority of the My units reacted at the early stages of the polymerization, considering the marked preference of the ⋯My˙ macro-radical for My (Fig. 1). This way, a My-rich segment including the middle block and the beginning of the two extended blocks may have been formed as well as two quasi neat P(IBOMA) outer blocks. The same hypothesis could be made for the IBOMA/S chain-extension, taking the higher reactivity of S for this system into account (*r*_S_ = 0.57, *r*_IBOMA_ = 0.20 reported with benzyl peroxide at 90 °C).^93^

**Fig. 8:**
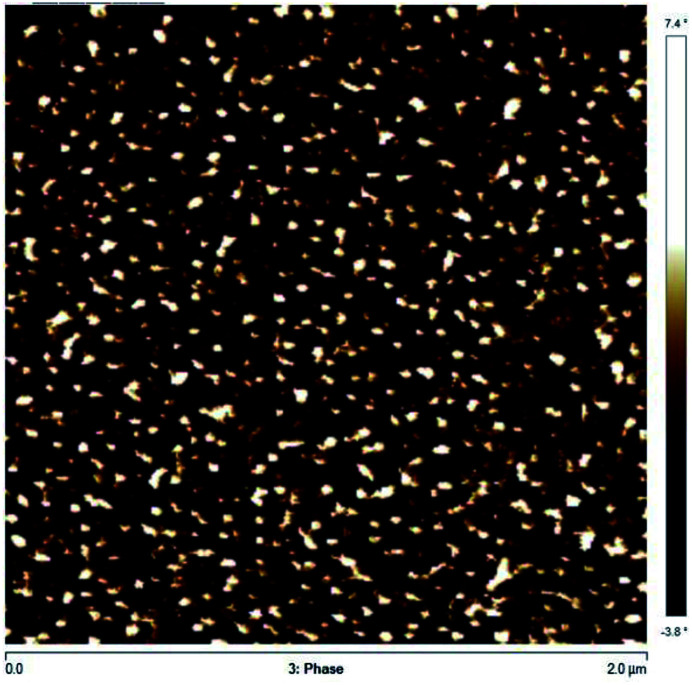
Atomic force microscopy (AFM, Experimental section) phase image (2 μm × 2 μm) under tapping mode of operation of the surface morphology of the triblock copolymer My-35-IBOMA/My cast film. The dark domain represents the My component (color-coded height scale given to the right of the image).

These DSC results suggest a two-phase system, due to the immiscibility between P(My) and IBOMA-rich segments. To further examine immiscibility, the solubility parameters *δ* were compared to estimate the tendency towards micro-phase separation. Using the group contribution method developed by van Krevelen and coworkers,^94^ theoretical solubility parameters for P(IBOMA) and 1,4-P(My) (favoured regioselectivity in our case) were determined (calculations can be found in ESI, page S16†) and *δ*_P(My)_ = 16.7 MPa^1/2^ and *δ*_P(IBOMA)_ = 21.8 MPa^1/2^ were calculated. Such a *δ* difference indicates a relatively high immiscibility between P(My) and P(IBOMA) segments and would suggest a strong bulk phase separation of P(My-*b*-IBOMA) block copolymer. Atomic force microscopy (AFM)^95^ was applied to the study of the surface morphology of My-35-IBOMA/My prepared by spin-coating a 3–4 wt% solution in chloroform. Fig. 8 shows a 2 μm × 2 μm scan of the polymeric surface (two other AFM phase images of My-35-IBOMA/My in different regions are given in ESI, Fig. S11†). The nature of the light and dark domains was determined by quantifying the relative contribution of the light component (ImageJ software application, binary mode). This image contained 23–24% of light areas which is consistent with the molar fraction of P(IBOMA) in the characterized triblock (*F*_IBOMA_ = 0.28, Table 4). The light areas can thus be attributed to P(IBOMA) and the dark ones to the rubbery P(My) midblock material. The phase separation of the two component blocks appeared clear with the embedding of glassy P(IBOMA) aggregates (disperse phase) in the soft P(My) (continuous phase). A spherical or cylindrical morphology might be obtained. An in-depth analysis, using for instance small-angle X-ray scattering (SAXS), would be required to ascertain the block copolymer morphology. It is of importance to note that the My-35-IBOMA/My self-assembly was disordered with a random location of the rigid aggregates, which exhibited various sizes (27% of large aggregates exhibiting a surface area of 2650 ± 740 nm^2^ and 73% of smaller aggregates having a surface area of 930 ± 480 nm^2^, quantification *via* Nanoscope Analysis software). By assuming that cylinders were observed (*F*_IBOMA_ = 0.28, possibly elevated for sphere formation), the radius of the cylinders *R* can be estimated according to the following formula (7):

7 *R* = 1.0*αKM*^1/2^

where *α* is the chain expansion parameter (*α* = 1.25 is generally assumed), *K* is the experimental constant related to the unperturbed root-mean-square end-to-end distance to the molecular weight (*K*_P(IBOMA)_ ≈ *K*_P(MMA)_ = 0.565 Å was assumed) and *M* = *M*_n,P(IBOMA)_ ≈ 16 kg mol^−1^ in this case.^96,97^ Accordingly, *R* ≈ 8.9 nm was estimated, suggesting that the surface area of the cylinders at the top/bottom (case where cylinders are perpendicular to AFM image, comparable to spheres) was equal to about 250 nm^2^. This may suggest that the relatively small aggregates seen on the AFM image (average surface area of 930 nm^2^) might not correspond to spheres but more likely to disoriented cylinders. For cylinders parallel to the AFM image having a mean length of 50 nm, a surface area of 2800 nm^2^ was calculated, which is comparable to the size of the large aggregates observed experimentally. Several factors may explain the asymmetrical domain organization. First, the presence of My units in the P(IBOMA) hard blocks resulted presumably in attractive interactions at the soft/hard interfaces, increasing the miscibility between the domains. The relatively broad molecular weight distribution of the sample (*Đ* = 1.91) may also lead to a reduction of the segregation strength (decrease in *χN*, where *χ* is the Flory–Huggins interaction parameter and *N* is the copolymer degree of polymerization).^98^ Lastly, the experimental approach can be discussed. The analyzed samples were not annealed prior to the AFM characterization. Indeed, due to the early thermal degradation of IBOMA-based polymers (Fig. 5), it was decided to not heat the samples at *T* > *T*_g,P(IBOMA)_ for a few hours or days. It may be argued that a stronger phase separation would have occurred after annealing, allowing a full relaxation of the P(IBOMA) chains.

Fig. 7b shows the temperature dependence of the shear storage and loss moduli (G′ and G′′ respectively) as well as the damping factor (tan *δ* = G′′/G′) from room temperature to 220 °C for the triblock copolymer My-52-IBOMA/S (Table 4C). The significant decrease of G′ from about 13 to 5 MPa with temperature until 70 °C can be first noted. A quasi constant G′ could have been expected in this temperature range due to the glassy state of the IBOMA units embedded in the soft rubbery P(My) phase. The presence of 12 mol% of S in the extended blocks can partly explain this behaviour. Compared to P(IBOMA), the solubility parameter of PS, *δ*_PS_ = 18.4 MPa^1/2^ (Hoftyzer and Van Krevelen approach), is closer to that of P(My), which may result in the partial homogeneity of the triblock copolymer. Moreover, short PS sequences, softening at relatively low temperature, were likely contained by the outer blocks. It was notably reported by DSC that *T*_g,PS_ = 32–69 °C for low *M*_n,PS_ = 0.9–3.0 kg mol^−1^.^99^ The rubbery plateau was then observed at *T* = 70–140 °C where G′ slightly decreased from 3.6 to 2.2 MPa. At *T* > 140 °C, the modulus dropped due to the softening of the P(IBOMA) phase, the flexible P(My) chains being no longer held together by the rigid domains. The maximum tan *δ* peak appeared at 171 °C, which is consistent with the P(IBOMA)'s *T*_g_ measured previously (*T*_g,P(IBOMA)_ = 171–177 °C, Fig. 4). Generally, this dynamic mechanical analysis (DMA) suggests that My-52-IBOMA/S may have an upper service temperature *T* ∼ 140 °C with G′ ≥ 2.2 MPa at *T* ≤ 140 °C.

**Fig. 9:**
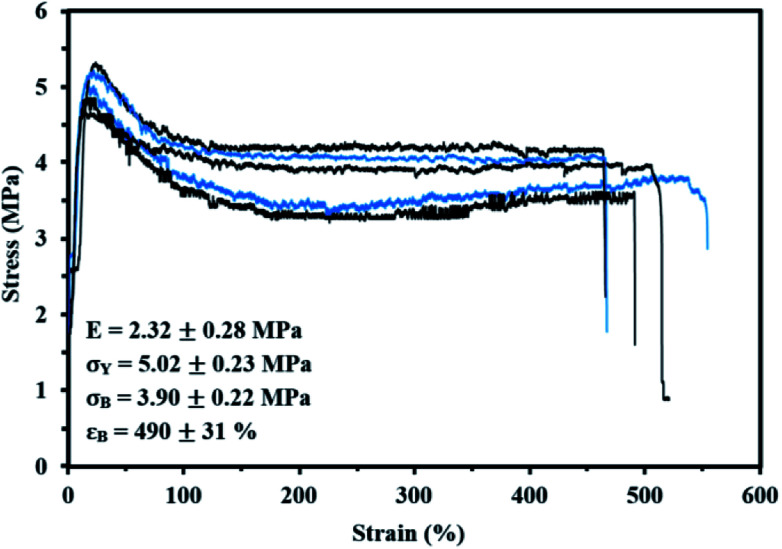
Tensile stress–strain curves of five My-52-IBOMA/S samples (same batch, color only used for differentiation) at room temperature and at a cross-head speed of 10 mm min^−1^. The average Young's modulus *E*, yield stress *σ*_Y_, tensile strength at break *σ*_B_ and tensile elongation at break *ε*_B_, obtained from these curves are also given.

Lastly, the mechanical properties at room temperature of My-52-IBOMA/S were determined by uniaxial tensile testing. The stress–strain curves are given in Fig. 9 along with the mean Young's modulus *E*, yield stress *σ*_Y_, tensile strength at break *σ*_B_ and tensile elongation at break *ε*_B_ values. The linear elastic region was apparent until 13–19% elongation, at which point the yield strength was observed. This low-strain region allowed the determination of *E* = 2.32 ± 0.28 MPa and *σ*_Y_ = 5.02 ± 0.23 MPa (Fig. 9). Beyond the proportionality limit, the tensile strength decreased (23% on average) until about 130% elongation. This can be presumably explained by the onset of the plastic deformation, not allowing the total recovery of the strains (permanent deformation of the material). The plastic region was then markedly observed with a plateau, characteristic of the drawing of the My-52-IBOMA/S chains, ending at the fracture point (*ε*_B_ = 490 ± 31%). It has to be noted that the strain hardening phenomenon, consisting of the orientation and alignment of polymer chains in the direction of the load which increases the strength and stiffness of the material, was not observed. Consequently, no increase in stress was measured at the fracture (*σ*_B_ = 3.90 ± 0.22 MPa). A key-factor allowing the understanding of this behaviour is presumably the morphology of the triblock copolymer. The possible disorganized structure of My-52-IBOMA/S, not containing necessarily P(IBOMA) glassy spheres can be detrimental. In this case, the expected physical crosslinking structure does not exist, bringing about a decrease of the entropic contribution to strain hardening. By assuming that the strain hardening process is associated with the debonding of the entanglement network (chain disentanglement), it may be also caused by the P(My) continuous phase, not being sufficiently entangled (*M*_n,MHS,My_ = 51.7 kg mol^−1^ < 3*M*_e,P(My)_),^85^ resulting in an unconstrained uncoiling of the chains. Generally, a flexible plastic was identified, having moderate elastic modulus and tensile strength at break combined with a good irreversible extensibility.

## Conclusions

My/IBOMA NMP in bulk at 100 °C initiated by NHS-BB highlighted the gradient nature of this copolymerization with the higher reactivity of the monoterpene (*r*_My_ = 1.90–2.16) toward both propagating species (⋯My˙ and ⋯IBOMA˙) compared of that of the bulky methacrylate (*r*_IBOMA_ = 0.02–0.07). While the homopolymerization of IBOMA was not controlled, likely due to the high equilibrium constant of this methacrylate, the addition of 20 mol% of My as a co-monomer allowed the synthesis of a well-defined P(My-grad-IBOMA) copolymer with *Đ* ≤ 1.39 and *M*_n,MHS_ close to the predicted one. More generally, well-controlled copolymerizations were performed for *f*_IBOMA,0_ ≤ 0.80 and the resulting P(My-grad-IBOMA)s exhibited *Đ* ≤ 1.41, *M*_n,MHS_ = 5.9–10.9 kg mol^−1^ and monomodal GPC distributions at overall conversion *X* = 25–65%. The deviations of experimental *M*_n,MHS_ values from theoretical ones were greater with *f*_My,0_, which may be caused by chain transfer side reactions to the monomers. Regardless of the initial feed composition, these copolymerizations had 〈*k*_p_〉〈*K*〉 = 2.0 ± 0.9 × 10^−5^ s^−1^ relatively close to that of My homopolymerization (1.4 ± 0.4 × 10^−5^ s^−1^), stressing the strong influence of My kinetics over the copolymerization kinetics. P(My-grad-IBOMA) displayed a glass transition temperature between −65 and +123 °C depending on the molar composition of IBOMA. A two-step thermal decomposition was observed by TGA, consisting of an early decomposition peak at *T*_dec,max_ = 242–307 °C, which results likely from the release of the isobornyl group, and a final decomposition peak at *T*_dec,max_ = 370–380 °C, due to the scission of the copolymer backbone. The chain-end fidelity of My-rich and IBOMA-rich P(My-grad-IBOMA)s was quantitatively deemed by ^31^P NMR (69–74% of SG1-terminated chains) and confirmed *via* successful chain-extensions in toluene with IBOMA and/or My (monomodal shift of the GPC traces).

The satisfactory mediation of the My/IBOMA system by the SG1 nitroxide allowed subsequently the preparation of triblock copolymers, composed of a soft P(My) middle block and two outer IBOMA-rich segments. 1,4-P(My)-(SG1)_2_ macro-initiator (*M*_n,MHS_ = 35–52 kg mol^−1^, *Đ* = 1.45–1.53) was first synthesized using the PEB-(SG1)_2_ dialkoxyamine initiator in bulk at 120 °C, followed by the IBOMA/Co chain-extension (Co = My or S co-monomer, <9 mol%) at 115 °C in toluene. Displaying two distinct *T*_g_s at −58 and +181 °C, My/IBOMA-My-IBOMA/My triblock copolymer (*M*_n,MHS_ = 51 kg mol^−1^, *Đ* = 1.91, *F*_IBOMA_ = 0.28) was studied by AFM, which revealed the micro-phase separation of the continuous P(My) domain and the disperse IBOMA-rich aggregates, despite an apparent disorganization. Lastly, rheological analysis as well as stress–strain tests were performed for the NMP-based S/IBOMA-My-IBOMA/S triblock copolymer (*M*_n,MHS_ = 95 kg mol^−1^, *Đ* = 2.23, *F*_IBOMA_ = 0.36, *F*_S_ = 0.05). In comparison to traditional styrenic block copolymers, an extended upper service temperature at around 140 °C was observed by DMA. Furthermore, this triblock copolymer exhibited a tensile strength at break *σ*_B_ ∼ 4 MPa and an elongation at break *ε*_B_ ∼ 500%, despite the absence of strain hardening.

## Conflicts of interest

There are no conflicts to declare.

## Supplementary Material

RA-009-C8RA09192G-s001
