# Supplementary material for: β-Myrcene/isobornyl methacrylate SG1 nitroxide-mediated controlled radical polymerization: synthesis and characterization of gradient, diblock and triblock copolymers
Source: RSC Adv. 2019 Jan 25;9(6):3377–95. doi: 10.1039/c8ra09192g (PMC9060242; doi:10.1039/c8ra09192g)
Supplement: RA-009-C8RA09192G-s001 [file RA-009-C8RA09192G-s001.pdf]

Electronic Supplementary Material (ESI) for RSC Advances.
This journal is © The Royal Society of Chemistry 2019

Supporting Information for:

β-Myrcene / Isobornyl Methacrylate SG1 Nitroxide-Mediated Controlled Radical Polymerization: Synthesis and Characterization of Gradient, Diblock and Triblock copolymers

Adrien Métafiot,[a,c,d] Lysandre Gagnon,[a] Sébastien Pruvost,[c] Pascal Hubert,[b] Jean-François Gérard,[c] Brigitte Defoort,[d] and Milan Marić[a]

[a]Department of Chemical Engineering, McGill University, 3610 University St., Montreal, H3A 2B2 Quebec, Canada

[b]Department of Mechanical Engineering, McGill University, 817 Sherbrooke St. W., Montreal, H3A 0C3 Quebec, Canada

[c]Ingénierie des Matériaux Polymères (IMP), CNRS UMR5223, INSA – Lyon, 17 Jean Capelle Avenue, 69621 Villeurbanne, France

[d]ArianeGroup, Avenue du Général Niox, 33160 Saint-Médard-en-Jalles, France

Keywords: *β*-myrcene; isobornyl methacrylate; nitroxide-mediated polymerization; copolymerization;

Corresponding Author: *E-mail: milan.maric@mcgill.ca (M.M.)

Contents of Supporting Information

Page

A) Preliminary polymerization temperature study **S3**
- Figure S1: $M_{n,MHS}$ and Đ *versus* overall conversion X for *My*/IBOMA copolymerization at 100 and 120 °C. **S3**

B) Determination of reactivity ratios by terminal models and a non-linear least-squares (NLLS) model **S4**
- Use of terminal models. **S4**
- Use of a NLLS model. **S4**
- Table S1: Samples used for determination of reactivity ratios. **S5**

C) Effect of feed composition on kinetics for *My*/IBOMA copolymerization **S6**
- Figure S2: $M_{n,GPC}$ and $M_{n,MHS}$ *versus* overall conversion X for experiments *My*/IBOMA-80 and *My*/IBOMA-30 at 100 °C. **S6**
- Figure S3: *ln*((1-X)$^{-1}$) *versus* *t* and linear trends for the *My*/IBOMA copolymerizations at 100 °C. **S7**
- Figure S4: *ln*((1-X)$^{-1}$) *versus* time *t*, Đ and $M_{n,MHS}$ *versus* X for experiments *My*/IBOMA-100, *My*/IBOMA-0 and *My*/IBOMA-0-Tol. **S8**

D) NMR spectra for P(*My-grad*-IBOMA) copolymers **S9**
- Figure S5: ^{1}H NMR spectrum of the final dry copolymer from experiment *My*/IBOMA-50. **S9**
- Figure S6: ^{31}P NMR spectra of macro-initiators *My*/IBOMA-82 and *My*/IBOMA-44. **S10**

E) DSC traces for P(*My-grad*-IBOMA) copolymers **S11**
- Figure S7: DSC traces for P(*My-grad*-IBOMA) gradient copolymers. **S11**

F) Optimized synthesis of P(*My*)-(SG1)$_2$ **S12**
- Table S2: Conditions for *My* polymerizations initiated by PEB-(SG1)$_2$.
- Figure S8: *ln*((1-X)$^{-1}$) *versus t*, Đ and $M_{n,MHS}$ *versus* X for experiments *My*-79, *My*-105, *My*-137 and *My*-174. **S12**
- Figure S9: *ln*((1-X)$^{-1}$) *versus t*, Đ and $M_{n,MHS}$ *versus* X for experiments *My*-174, *My*-173$_{SG1,9}$, *My*-169$_{SG1,18}$ and *My*-170$_{Tol}$. **S13**

S14

G) IBOMA/Co chain-extension from P(*My*)-(SG1)$_2$ **S15**
- Figure S10: Normalized GPC traces for the chain-extensions of P(*My*)-(SG1)$_2$ with IBOMA/*My* and IBOMA/S mixtures **S15**

H) Characterization of the Co/IBOMA-*My*-IBOMA/Co triblock copolymers **S16**
- Solubility parameter via the method of Hoftyzer and Van Krevelen **S16**
- Figure S11: AFM phase images of *My*-35-IBOMA/*My* **S17**
- Figure S12: Shear storage and loss moduli *versus* strain for *My*-52-IBOMA/S **S18**

A) Preliminary polymerization temperature study

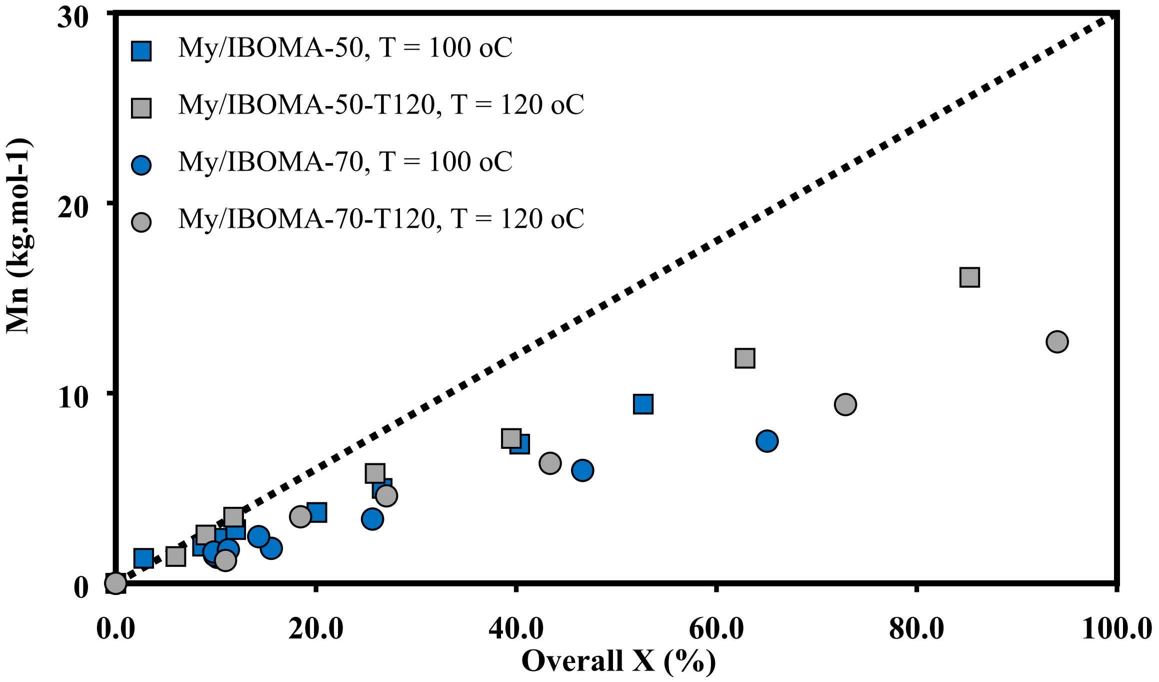

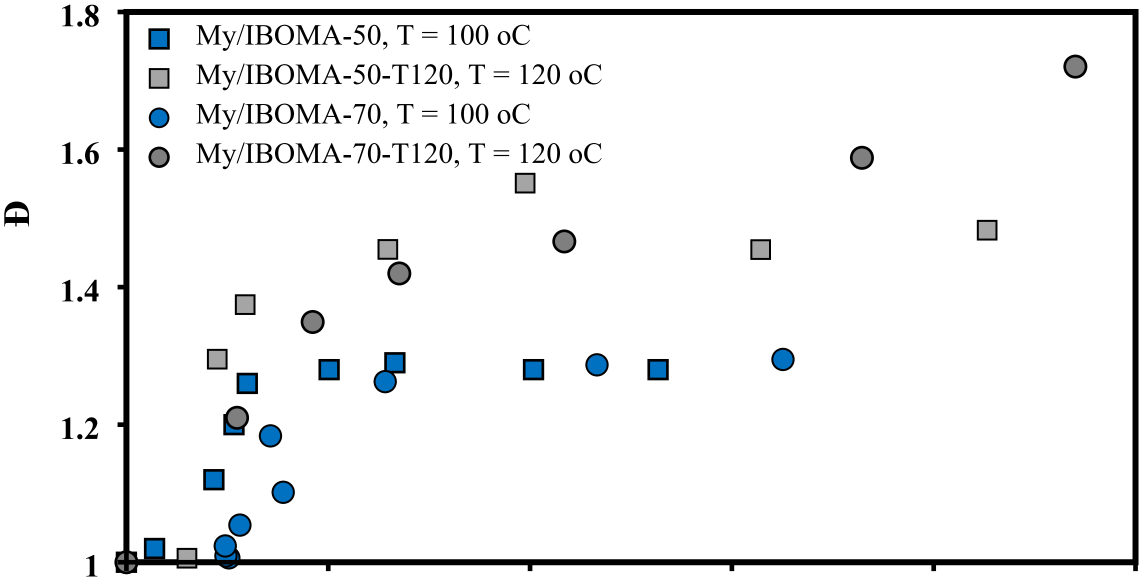

Figure S1. $M_{n,MHS}$ determined by GPC relative to PMMA standards in THF at 40 °C and corrected via MHS parameters *versus* overall conversion X (**top**, the dotted line indicates the theoretical M_n ($M_{n,theo}$) *versus* overall conversion based on the monomers to initiator ratio) and Đ *versus* overall conversion X (**bottom**) for *My*/IBOMA copolymerizations performed at T = 100-120 °C, and exhibiting $f_{My,0}$ = 0.50-0.71.

B) Determination of reactivity ratios by terminal models and a non-linear least-squares (NLLS) model

◘ **Use of terminal models:** Table S1 gives the experimental data used to determine the reactivity ratios r_{My} and r_{IBOMA}. It was assumed that the selected copolymer samples exhibited sufficiently a low degree of conversion that the monomer composition was essentially unchanged. Calculation of r_{My} and r_{IBOMA} was first done by the Fineman-Ross (FR) approach[1] (rearrangement of the Mayo-Lewis copolymer equation into a linear form[2]) and by the Kelen-Tüdös (KT) approach[3]. The latter refines the linearization method by introducing an arbitrary positive constant α to spread the data more evenly so as to give equal weight to all data points. Accordingly, the KT approach minimizes the bias of the FR approach. The appropriate plot of the variables G and H, defined in Equation B1, will provide a linear relationship giving the reactivity ratios as the slope and intercept as shown in Equation B2.

$G = (f_{IBOMA,0} / f_{My,0}) \times [(2F_{IBOMA} - 1) / F_{IBOMA}]$; $H = (f_{IBOMA,0} / f_{My,0})^2 \times [(1 - F_{IBOMA}) / F_{IBOMA})$ **(B1)**

$G = r_{IBOMA} \times H - r_{My}$ **(B2)**

Equation B3 shows the additional variables used for the KT plot as illustrated in Equation B4.

$\eta = G / (\alpha + H)$; $\varepsilon = H / (\alpha + H)$; $\alpha = \sqrt{H_{max} H_{min}}$ **(B3)**

$\eta = (r_{IBOMA} + r_{My} / \alpha)\varepsilon - r_{My} / \alpha$ **(B4)**

In Equations B3 and B4, H_{max} and H_{min} stand for the highest and lowest values of the set of experimental points, respectively. $r_{My} = 2.16 \pm 0.34$ and $r_{IBOMA} = 0.07 \pm 0.04$ ($r_{My} \times r_{IBOMA} = 0.15 \pm 0.19$) were obtained via the FR method and $r_{My} = 1.90 \pm 0.18$ and $r_{IBOMA} = 0.02 \pm 0.21$ ($r_{My} \times r_{IBOMA} = 0.04 \pm 0.15$) were calculated via the KT method. The errors associated with the experimental data were derived from the standard errors of the slopes from FR and KT plots.

◘ **Use of a NLLS model:** Despite its improvements, the KT method does not overcome the shortcomings of the linear method: use of the differential form and errors that affect both variables. In order to handle rigorously the Mayo-Lewis equation, linearization has to be avoided. From a statistical point of view, a NLLS fit to the Mayo-Lewis equation is probably the soundest method to determine the desired parameters[4]. Therefore, the reactivity ratios were also determined using a non-linear least-squares fitting of the data. The commercial software package Matlab R2016a was used to solve the Mayo-Lewis equation[1] for *My*/IBOMA copolymerization (B5).

$$F_{IBOMA} = (r_{IBOMA} f_{IBOMA,0}^2 + f_{IBOMA,0} f_{My,0}) / (r_{IBOMA} f_{IBOMA,0}^2 + 2 f_{IBOMA,0} f_{My,0} + r_{My} f_{My,0}^2) \quad \textbf{(B5)}$$

Using the reactivity ratios determined by the KT method as initial guesses, the statistical fit to the data yielded reactivity ratios $r_{My} = 2.07 \pm 0.58$ and $r_{IBOMA} = 0.05 \pm$

0.08 at 95 % confidence level and with a regression coefficient $R^2 = 0.91$ (SSE = 0.0174, RMSE = 0.0499).

Table S1. Samples used for the determination of reactivity ratios for *My*/IBOMA gradient copolymerization done at 100 °C in bulk with NHS-BB and targeting $M_{n,theo} = 30$ kg.mol^{-1} at X = 100 %.

$f_{IBOMA,0}$ [(a)]	F_{IBOMA} [(b)]	X[(c)] (%)	*t* (min)	$M_{n,MHS}$ [(d)] (kg.mol^{-1})	Đ[(d)]
0.10	0.15	24.5	60	4.1	1.27
0.19	0.10	12.5	30	1.6	1.07
0.30	0.13	13.2	60	1.8	1.10
0.41	0.22	17.0	120	2.7	1.27
0.50	0.24	20.1	100	3.7	1.28
0.60	0.28	10.4	30	3.0	1.21
0.70	0.42	13.2	60	3.5	1.30
0.80	0.39	21.2	180	6.6	1.36
0.90	0.57	12.4	60	7.0	1.52

a) $f_{IBOMA,0}$ is the initial molar feed composition of isobornyl methacrylate.

b) F_{IBOMA} is the molar composition of isobornyl methacrylate in the P(*My*-*grad*-IBOMA) copolymer determined by ^{1}H NMR in $CDCl_3$.

c) Overall monomer conversion $X = X_{My} f_{My,0} + X_{IBOMA} f_{IBOMA,0}$ determined by ^{1}H NMR in $CDCl_3$.

d) $M_{n,GPC}$ and $M_{w,GPC}$ determined by GPC calibrated with PMMA standards in THF at 40 °C. $M_{n,MHS}$, obtained from $M_{n,GPC}$ and corrected using the Mark-Houwink relationship.

C) Effect of feed composition on kinetics for *My*/IBOMA copolymerization

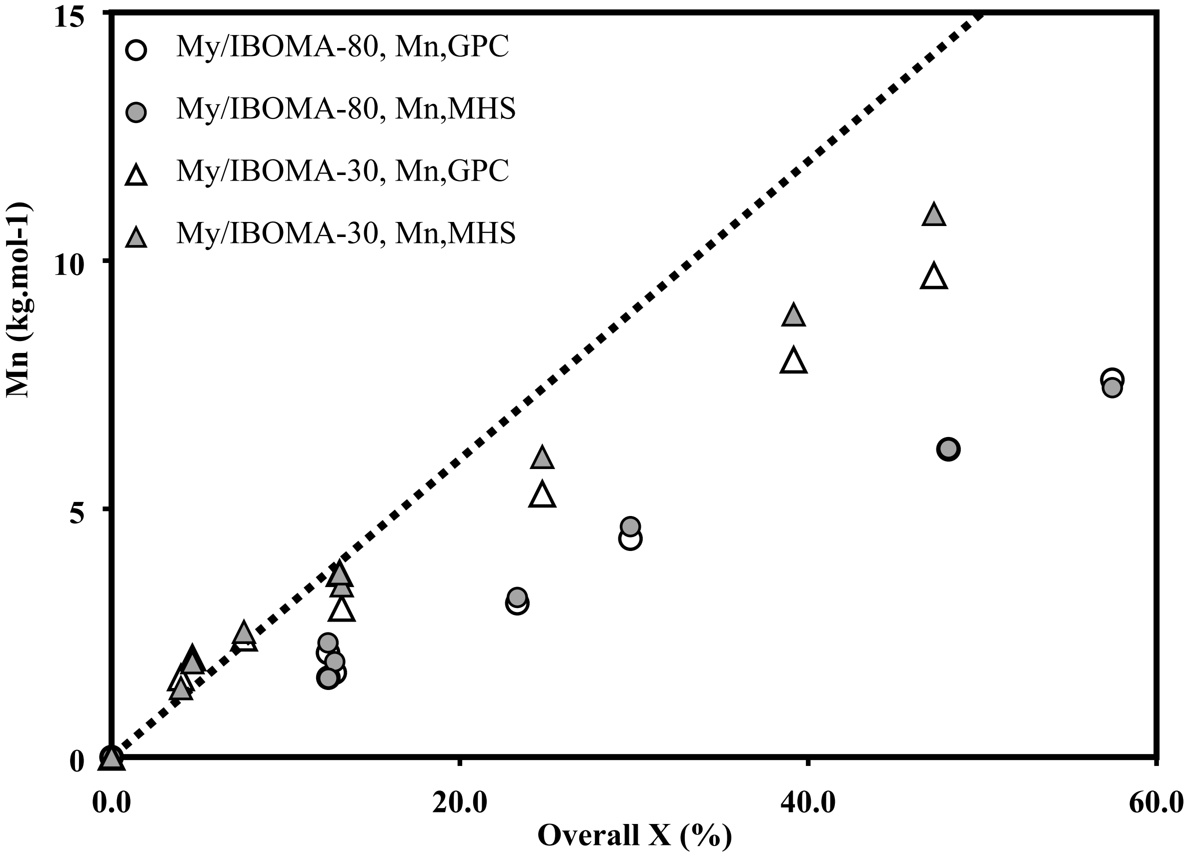

Figure S2. $M_{n,GPC}$ determined by GPC relative to PMMA standards in THF at 40 °C and $M_{n,MHS}$, obtained from $M_{n,GPC}$ and corrected using the Mark-Houwink relationship, *versus* overall conversion X for experiments *My*/IBOMA-80 ($f_{My,0}$ = 0.81) and *My*/IBOMA-30 ($f_{My,0}$ = 0.30) at 100 °C. The dotted line indicates the theoretical M_n ($M_{n,theo}$) *versus* overall conversion based on the monomers to initiator ratio ($M_{n,theo}$ = 30 kg.mol^{-1} at X = 100%).

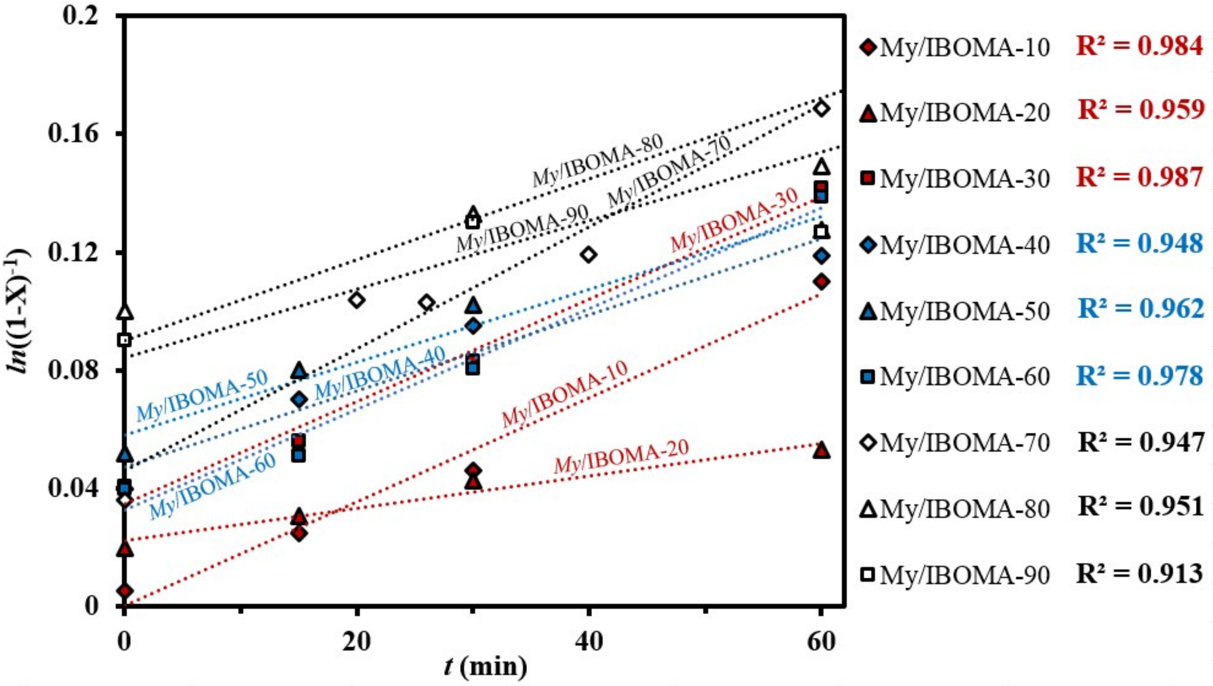

versus polymerization time *t* for the various *My*/IBOMA copolymerizations in bulk at 100 °C initiated by NHS-BB. All experimental ID and characterization of experiments are listed in Table 1 and Table 2 of the manuscript. The dotted lines indicate linear fits to the experimental data during the initial stages of the polymerizations (0 to 60 min, except for *My*/IBOMA-80 and *My*/IBOMA-90 where the linear region was taken from 0 to 120 min). The squared linear regression coefficient R^2 for each linear trend is also provided.

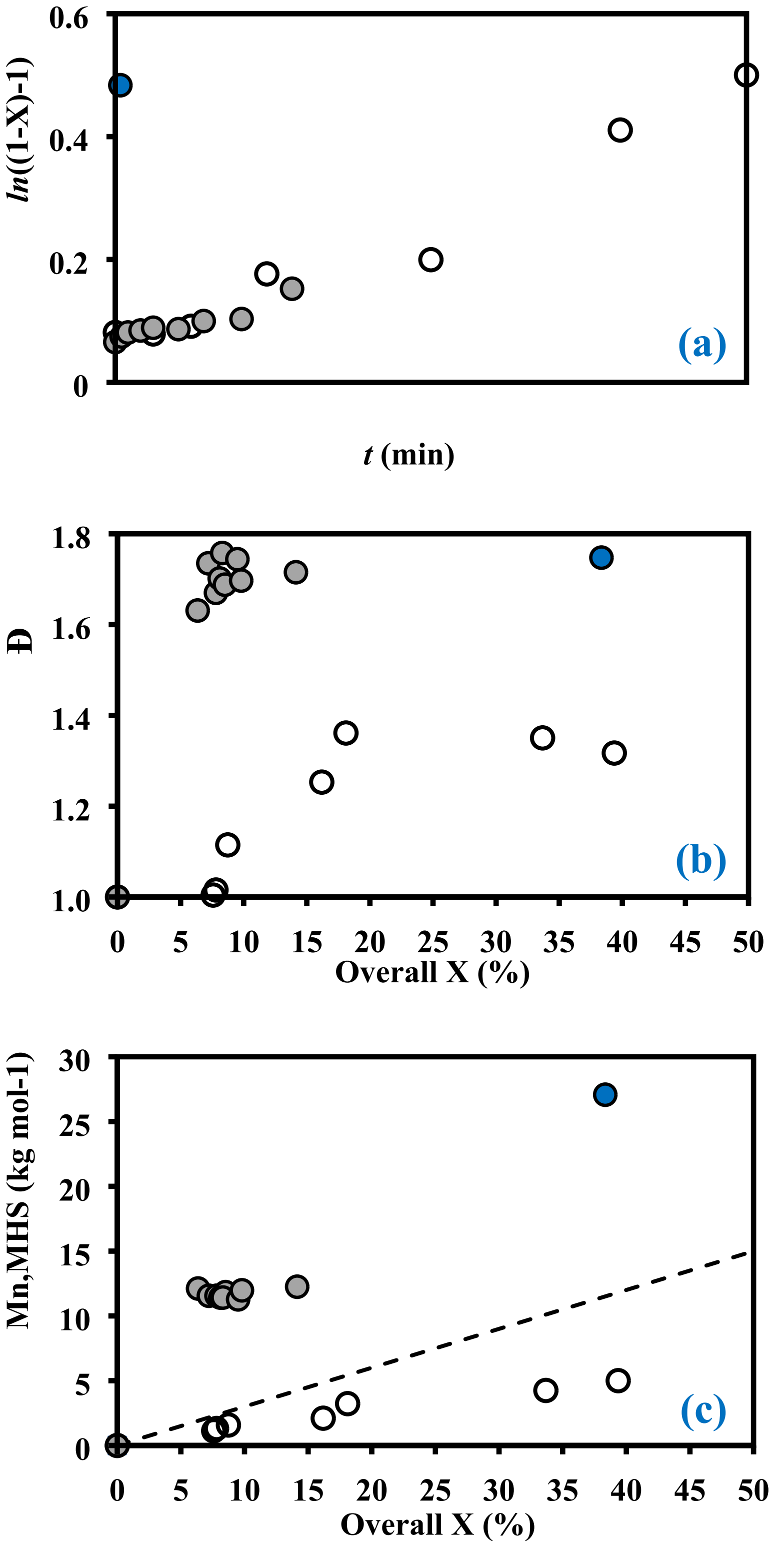

Figure S4. **(a)** *ln*($(1 - X)^{-1}$) (X = overall conversion) *versus* reaction time *t*, **(b)** Đ *versus* overall conversion X and **(c)** $M_{n,MHS}$ *versus* overall conversion X for *My*/IBOMA-100 ($f_{My,0}$ = 1.00, open circles (○)), *My*/IBOMA-0 ($f_{My,0}$ = 0, solid blue circles (●)) and *My*/IBOMA-0-Tol ($f_{My,0}$ = 0, in toluene, solid grey circles (●)). The dashed line indicates the theoretical M_n *versus* overall conversion based on the monomer

D) NMR spectra for P(*My*-*grad*-IBOMA) copolymers

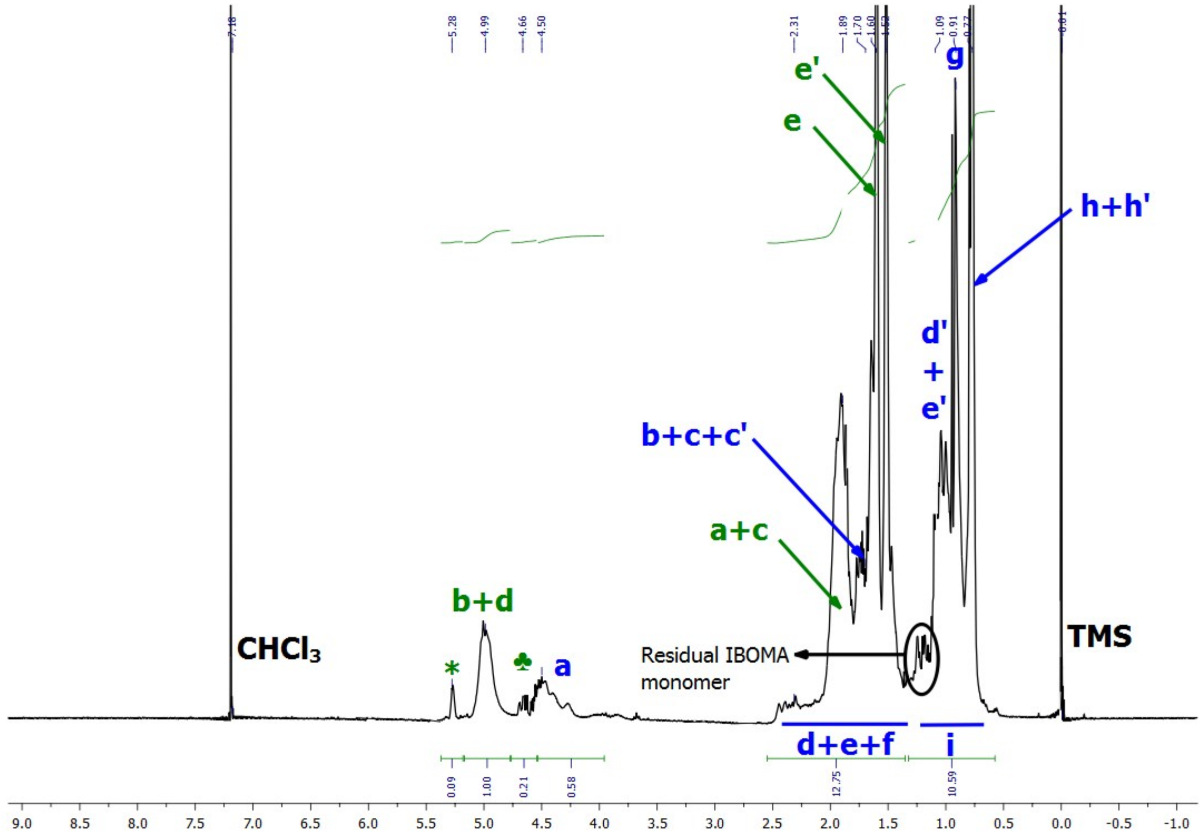

P(*My*-*grad*-IBOMA) *My*/IBOMA-50: ^{1}H NMR ($CDCl_3$, 300 MHz, RT): δ = 5.20-4.90 (s br,

Figure S5. ^{1}H NMR spectrum in $CDCl_3$ (300 MHz, tetramethylsilane TMS as internal reference) at room temperature of the final dried P(*My*-*grad*-IBOMA) gradient copolymer from experiment *My*/IBOMA-50 after recovery and purification (dissolution via tetrahydrofuran and precipitation via methanol).

2H$^{P(My)}$), 4.65-4.00 (m br, 1H$^{P(IBOMA)}$), 2.60-1.40 (m br, 7H$^{P(IBOMA)}$), 2.20-1.90 (m br, 8H$^{P(My)}$), 1.67 (s, 3H$^{P(My)}$), 1.59 (s, 3H$^{P(My)}$), 1,40-0.70 (m br, 14H$^{P(IBOMA)}$).

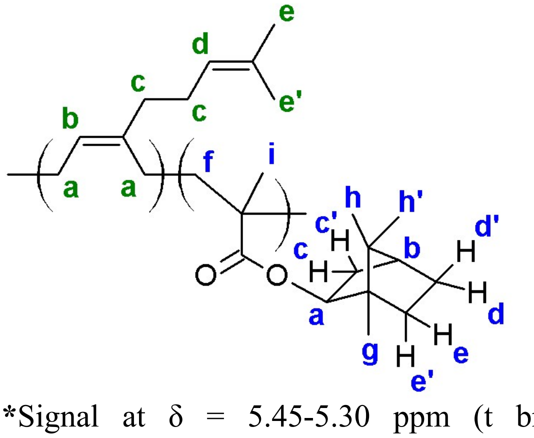

1,2-

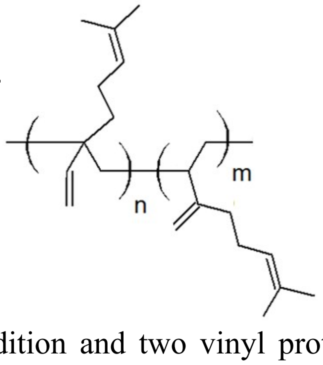

tent

*Signal at δ = 5.45-5.30 ppm (t br, 1H$^{1,2\text{-}P(My)}$) corresponds to one olefinic proton of 1,2-P(*My*)[5].

♣Signal at δ = 4.80-4.65 ppm (d, 2H$^{1,2\text{-}P(My)}$ and s, 2H$^{3,4\text{-}P(My)}$) corresponds to two vinyl protons of 3,4-addition and two vinyl protons of 1,2-addition of *My* units[5].

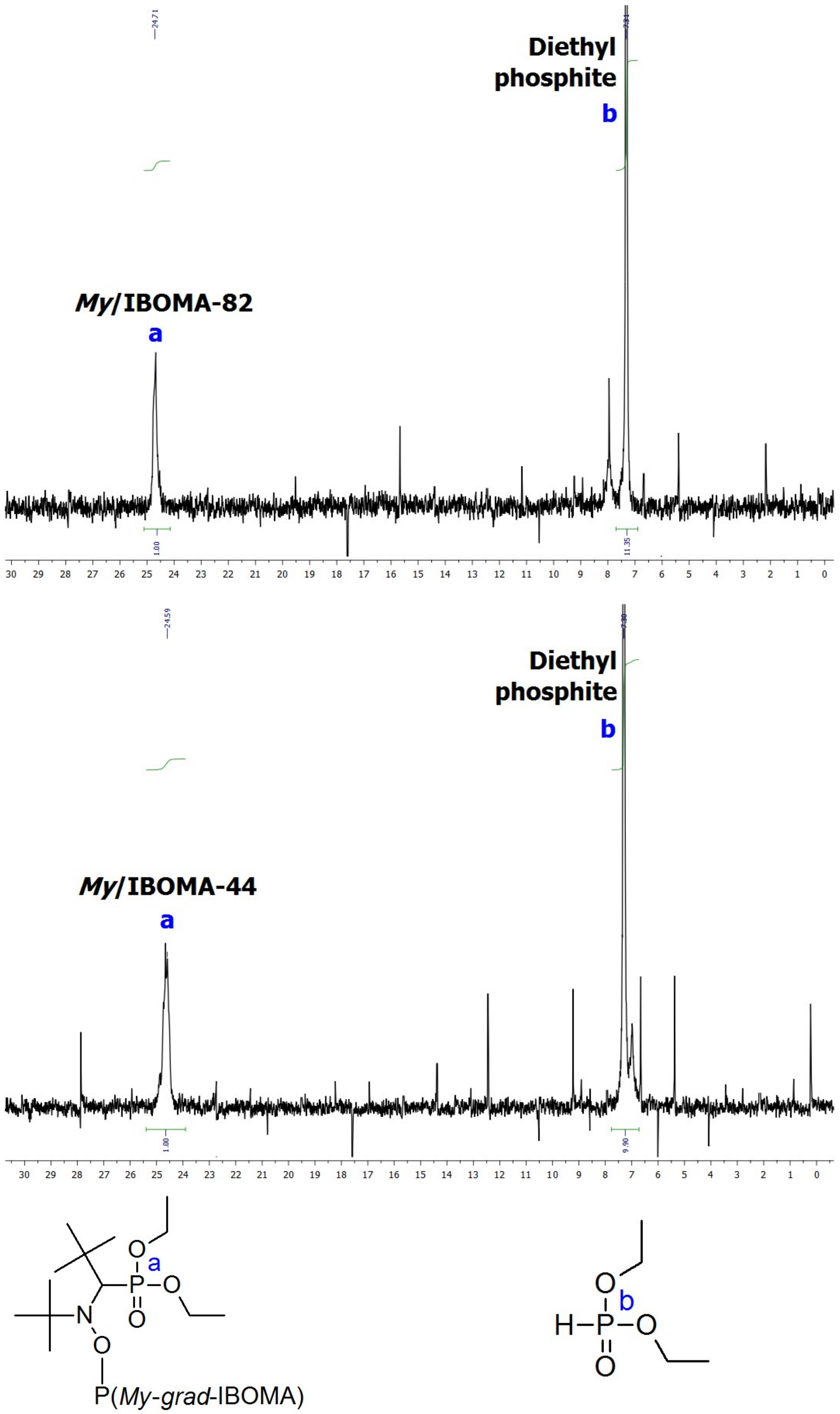

Figure S6. 81 MHz ^{31}P NMR spectra of dried P(*My-grad*-IBOMA)-SG1 macroinitiators *My*/IBOMA-82 (top) and *My*/IBOMA-44 (down, chemical structure in bottom left) in $CDCl_3$ at room temperature with diethyl phosphite as an internal reference (structure in bottom right).

E) DSC traces for P(*My*-*grad*-IBOMA) copolymers

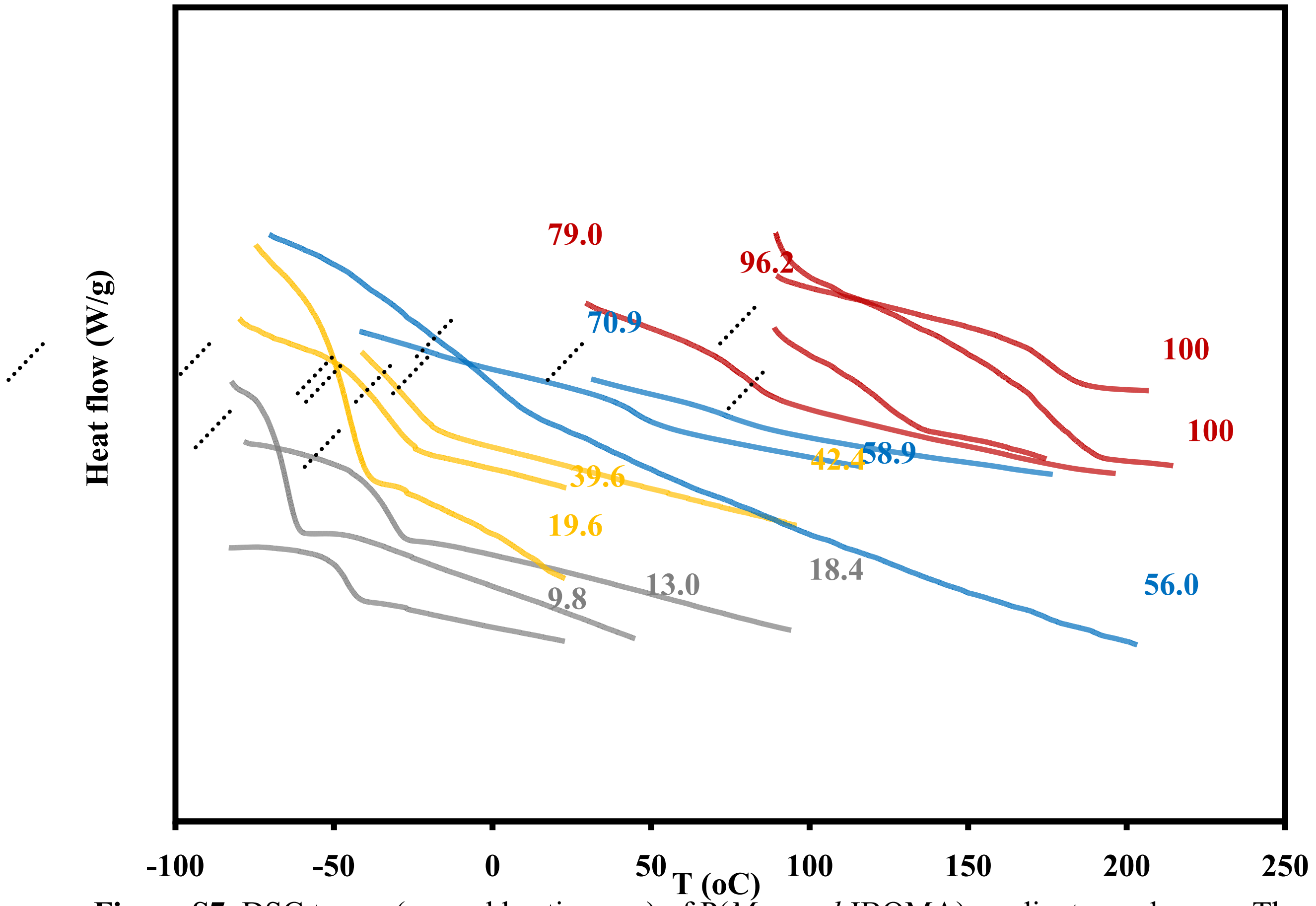

Figure S7. DSC traces (second heating run) of P(*My*-*grad*-IBOMA) gradient copolymers. The black dotted lines indicate the changes in slope observed. The numbers alongside each DSC curve refer to the molar fraction of IBOMA (F_{IBOMA} in percentage) in the copolymer. Please note that the P(*My*-*grad*-IBOMA) copolymers characterized by DSC do not necessarily correspond to the final copolymers presented in Table 2.

F) Optimized synthesis of P(*My*)-(SG1)$_2$

Table S2. Experimental conditions for *My* polymerizations initiated by PEB-$(SG1)_2$ at 120 ^oC in bulk or at 115 ^oC in toluene.

ID[(a)]	[PEB-$(SG1)_2]_0$ (M)	$[My]_0$ (M)	$[SG1]_0$ (M)	Solvent	$[Solvent]_0$ (M)	$M_{n,theo}$[(b)] (kg.mol^{-1})	*t* (min)
***My*-79**	0.011	5.928	0	-	0	79.1	300
***My*-105**	0.008	5.831	0	-	0	105.0	420
***My*-137**	0.006	5.791	0	-	0	137.2	420
***My*-174**	0.005	6.178	0	-	0	174.0	420
***My*-173$_{SG1,9}$**	0.005	6.122	0.0005	-	0	172.5	420
***My*-169$_{SG1,18}$**	0.005	5.975	0.0011	-	0	168.5	480

a) Experimental identification given by *My*-XX where XX refers to the rounded $M_{n,theo}$ targeted.

b) given for X = 100% and calculated as follows: $M_{n,theo} = M_{My}$ ($[My]_0$ /[PEB-$(SG1)_2]_0$) + $M_{n,PEB-(SG1)2}$ with M_{My} = 136.23 g.mol^{-1} and $M_{n,PEB-(SG1)2}$ = 5.7 kg.mol^{-1}.

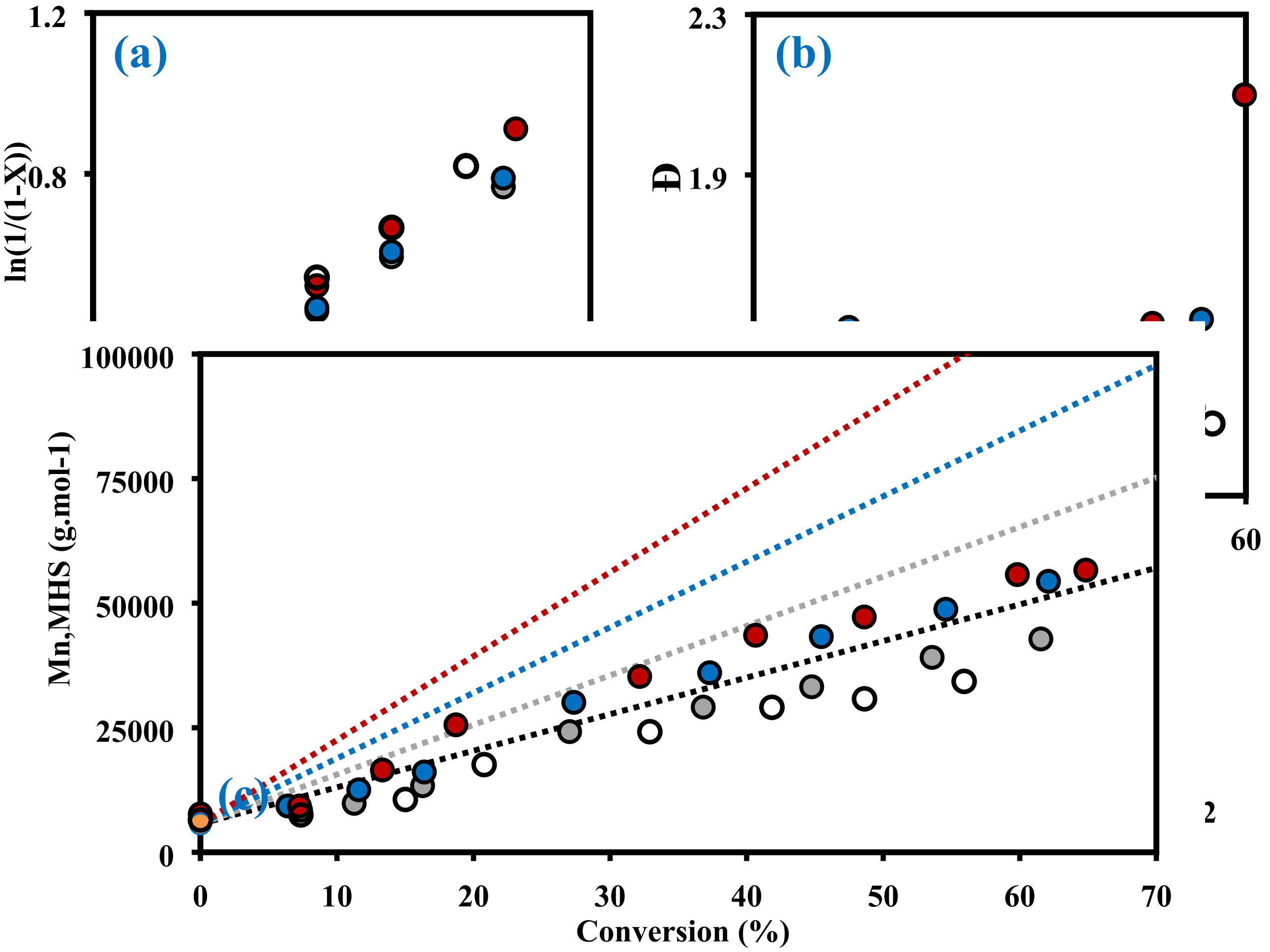

Figure S8. a) Semi-logarithmic kinetic plots of $ln((1-X)^{-1})$ (X = *My* conversion) *versus* polymerization time *t*, (**b**) Đ *versus* X, and (**c**) $M_{n,MHS}$ *versus* X for the experiments *My*-79 (open circles), *My*-105 (solid grey circles), *My*-137 (solid blue circles) and *My*-174 (solid red circles). The dotted lines indicate the theoretical M_n *versus* overall conversion based on the monomer-to-initiator ratio for each reaction (black, grey, blue and red predicted lines for experiments *My*-79, *My*-105, *My*-137 and *My*-174 respectively). All experimental ID are listed in Tables S2 above.

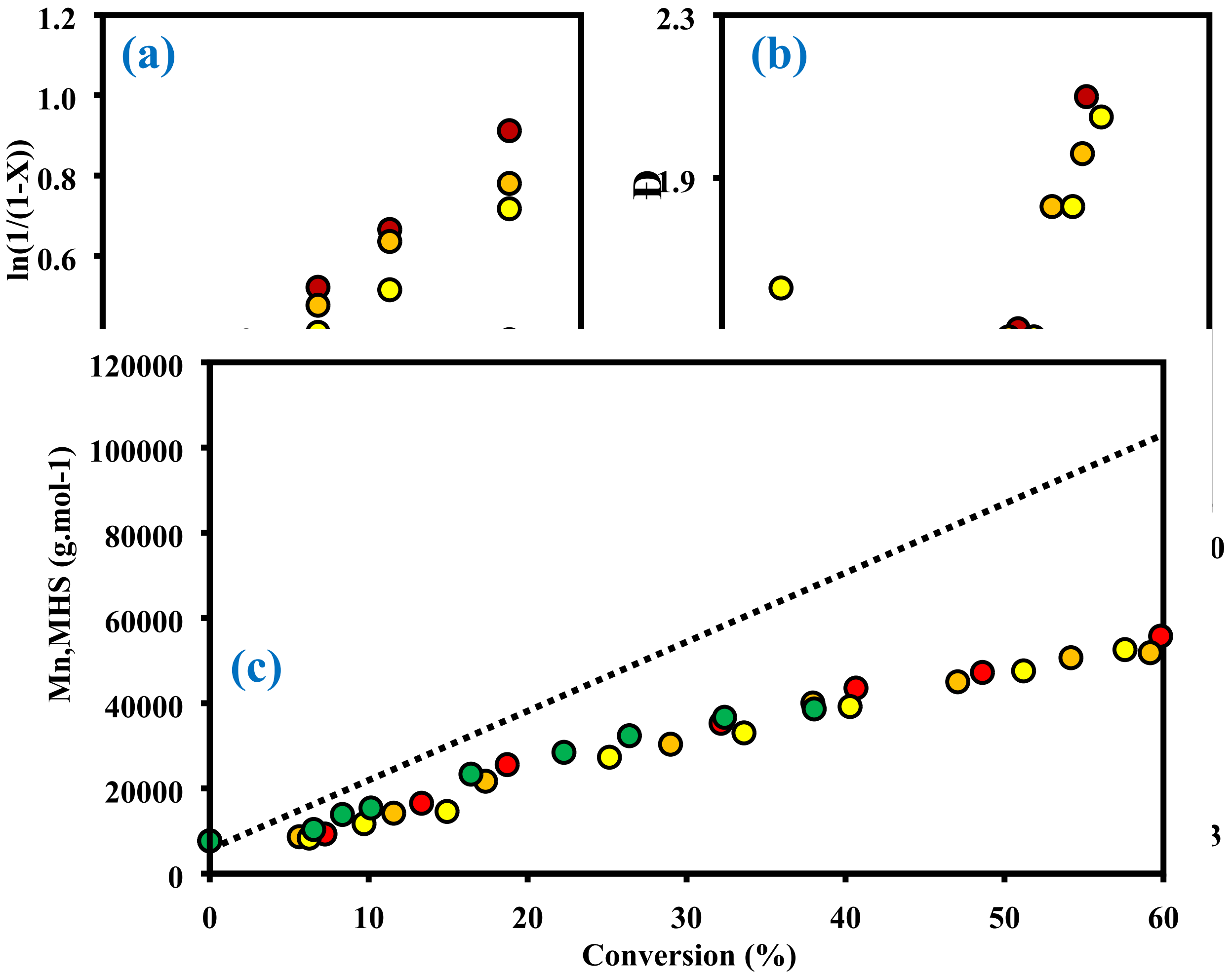

Figure S9. a) Semi-logarithmic kinetic plots of $ln((1-X)^{-1})$ (X = *My* conversion) *versus* polymerization time *t*, (**b**) Đ *versus* X, and (**c**) $M_{n,MHS}$ *versus* X for the experiments *My*-174 (solid red circles), *My*-$173_{SG1,9}$ (solid orange circles), *My*-$169_{SG1,18}$ (solid yellow circles) and *My*-170_{Tol} (solid green circles). The dotted line indicates the average theoretical M_n *versus* overall conversion based on the monomer-to-initiator ratio (average $M_{n,theo}$ = 171.3 kg.mol^{-1} at X = 1.0). All experimental ID are listed in Tables S2 above.

G) IBOMA/Co chain-extension from P(*My*)-$(SG1)_2$

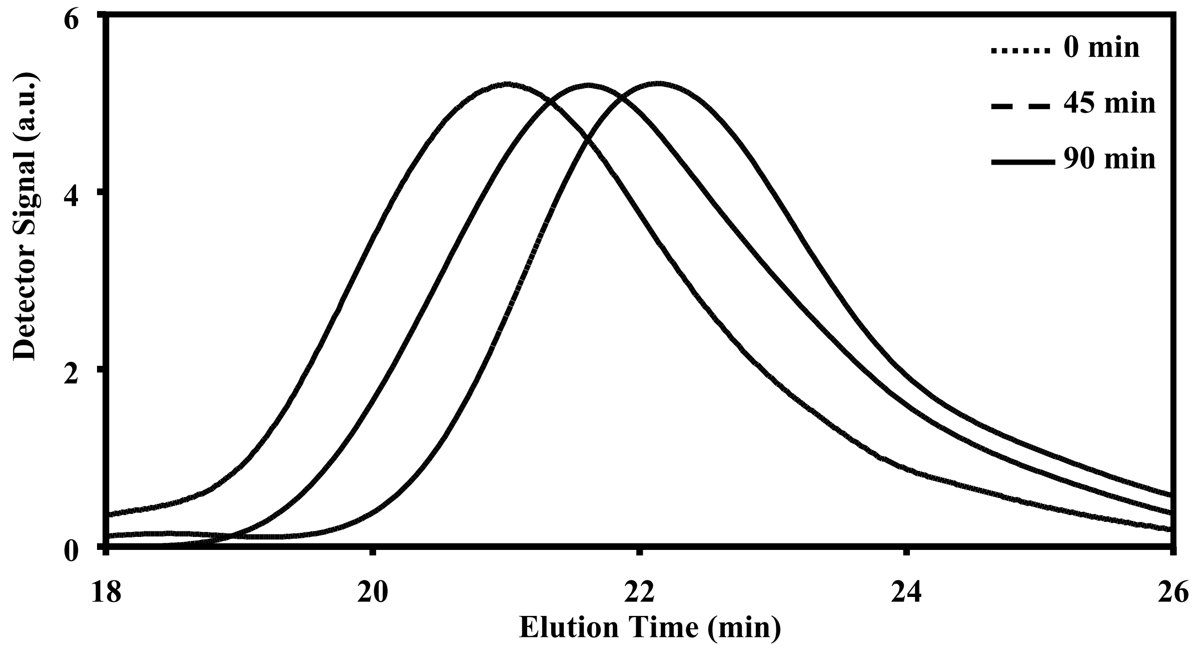

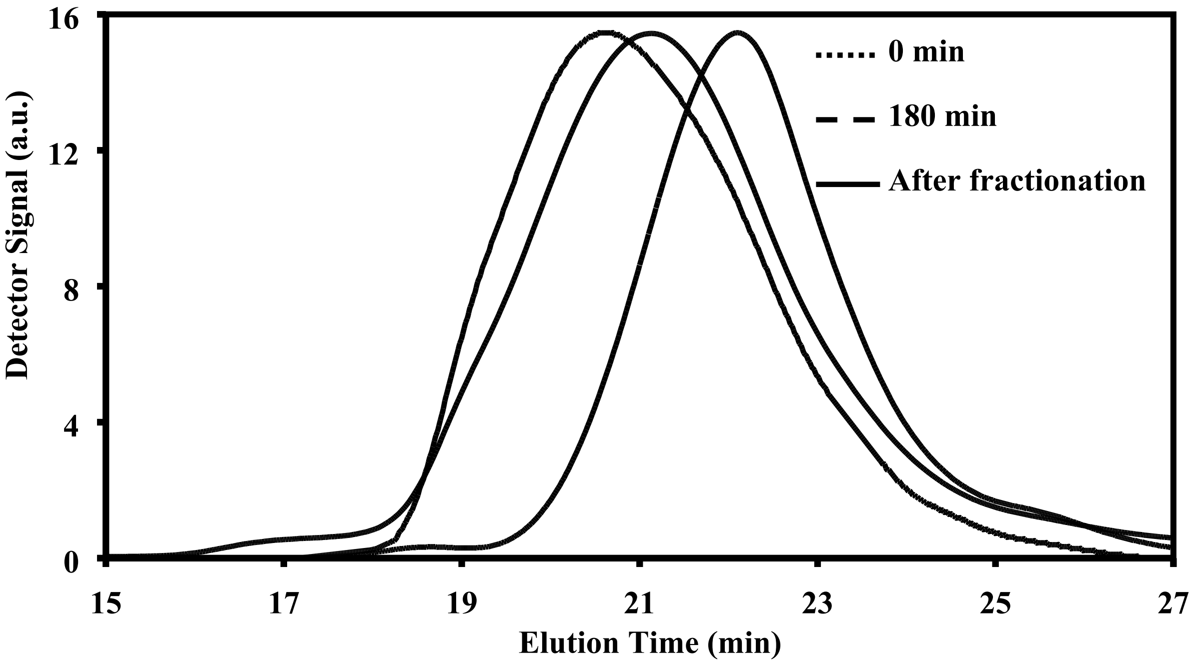

H) Characterization of the NMP-based Co/IBOMA-*My*-IBOMA/Co triblock copolymers

Figure S10. Normalized GPC traces for the chain-extensions of **a)** *My*-35 with a IBOMA/*My* (92/8 mol%) mixture (experiment *My*-35-IBOMA/*My*) and **b)** *My*-52 with a IBOMA/S (91/9 mol%) mixture (experiment *My*-52-IBOMA/S) at T = 115 °C in 50 wt% toluene. For the experiment *My*-52-IBOMA/S, a slight shoulder can be observed (elution time = 16-18 min) for the sample taken at 180 min. This may be a result of termination by combination, characterized by a coupling of two propagating radicals. Fractionation of the final *My*-52-IBOMA/S sample was performed using a methanol (non-solvent) / benzene (solvent) pair (2 cycles).

▫ **Calculation of theoretical solubility parameter via the method of Hoftyzer and Van Krevelen[6]:** Relying on the group contribution principle, the solubility parameter components can be determined using the following equations:

$$\delta_d = \sum F_{di} / V_m \quad ; \quad \delta_p = (\sum F_{pi}^2)^{0.5} / V_m \quad ; \quad \delta_h = (\sum E_{hi} / V_m)^{0.5} \qquad \textbf{(H1)}$$

where δ_d, δ_p and δ_h are the contributions of dispersion forces, polar forces and hydrogen bonding, respectively. F_{di} and F_{pi} are the molar attraction constants for dispersion and polar forces. E_{hi} is the cohesive energy for hydrogen bonding and V_m is the molar volume of the structural unit of the polymer. The corresponding equation for determination of solubility parameter (δ) is the following:

$$\delta = (\delta_d^2 + \delta_p^2 + \delta_h^2)^{0.5} \qquad \textbf{(H2)}$$

▫ **Solubility parameter of 1,4-P(*My*):** Addition of the group contributions for 1,4-P(*My*) gives:

	F_{di} ($MPa^{1/2}.mol^{-1}$)	F_{pi}^2 ($MPa.mol^{-2}$)	E_{hi} ($J.mol^{-1}$)	V_m ($cm^3.mol^{-1}$)
2 $–CH_3$	+ 420	0	0	+ 33.5
4 $–CH_2$-	+ 270	0	0	+ 16.1
2 =CH-	+ 200	0	0	+ 13.5
2 =C<	+ 70	0	0	- 5.5
Total	2 460	0	0	147.4

Thus: $\delta_{P(My)} = (16.69^2 + 0^2 + 0^2)^{0.5} = 16.69\ MPa^{1/2}$

▫ **Solubility parameter of P(IBOMA):** Addition of the group contributions for P(IBOMA) gives:

	F_{di} ($MPa^{1/2}.mol^{-1}$)	F_{pi}^2 ($MPa.mol^{-2}$)	E_{hi} ($J.mol^{-1}$)	V_m ($cm^3.mol^{-1}$)
4 $–CH_3$	+ 420	0	0	+ 33.5
4 $–CH_2$-	+ 270	0	0	+ 16.1
3 >C<	- 70	0	0	- 19.2
2 >CH-	+ 80	0	0	- 1.0
1 –CO-	+ 290	+ 592 900	+ 2 000	+ 10.8
1 –O-	+ 100	+ 160 000	+ 3 000	+ 3.8
Total	3 100	752 900	5 000	153.4

Thus: $\delta_{P(IBOMA)} = (20.21^2 + 5.66^2 + 5.71^2)^{0.5} = 21.75\ MPa^{1/2}$

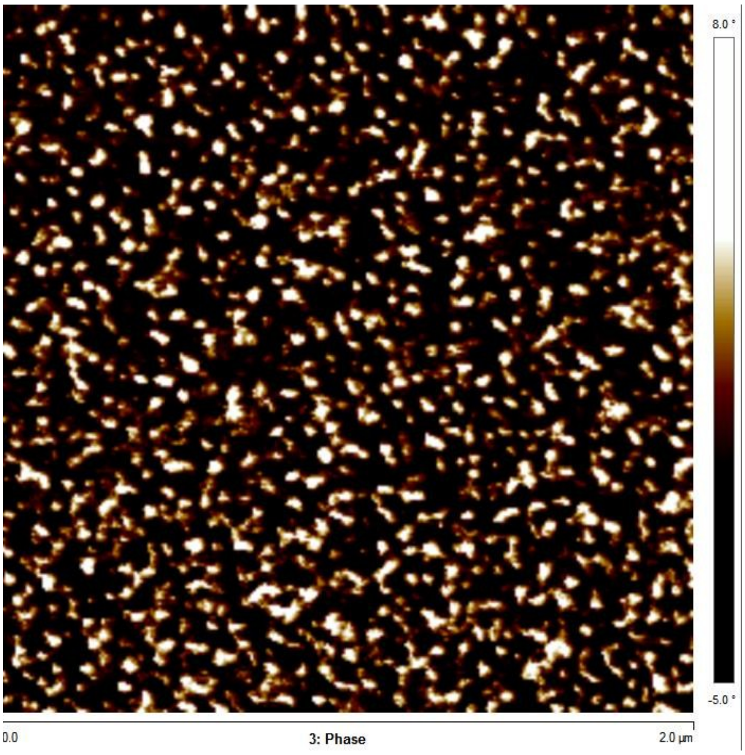

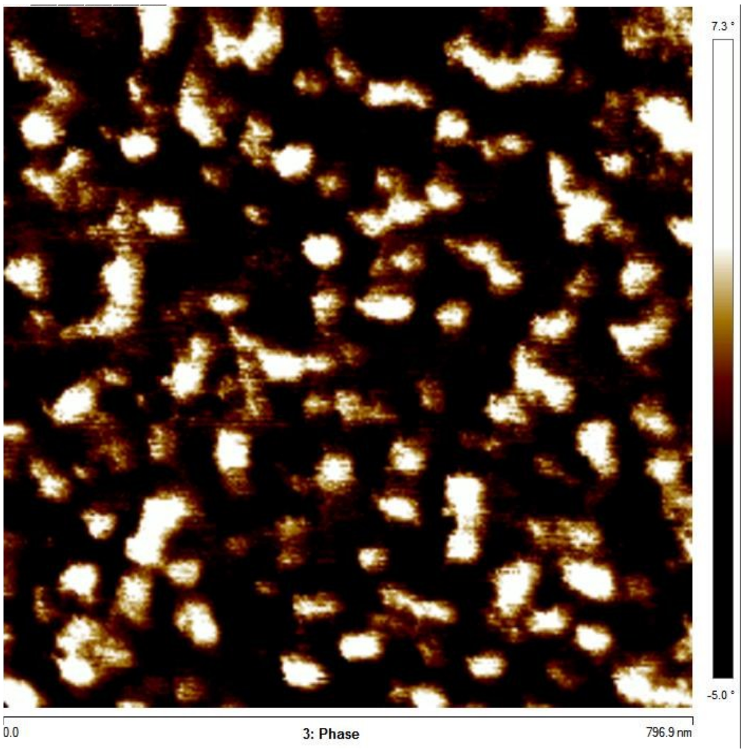

Figure S11. Atomic force microscopy phase images (top image, 2 µm x 2 µm; bottom image, 0.8 µm x 0.8 µm) under tapping mode of operation of the surface morphology of the triblock copolymer *My*-35-IBOMA/*My* cast film**.** The dark domain represents the *My* component (color-coded height scale given to the right of the image).

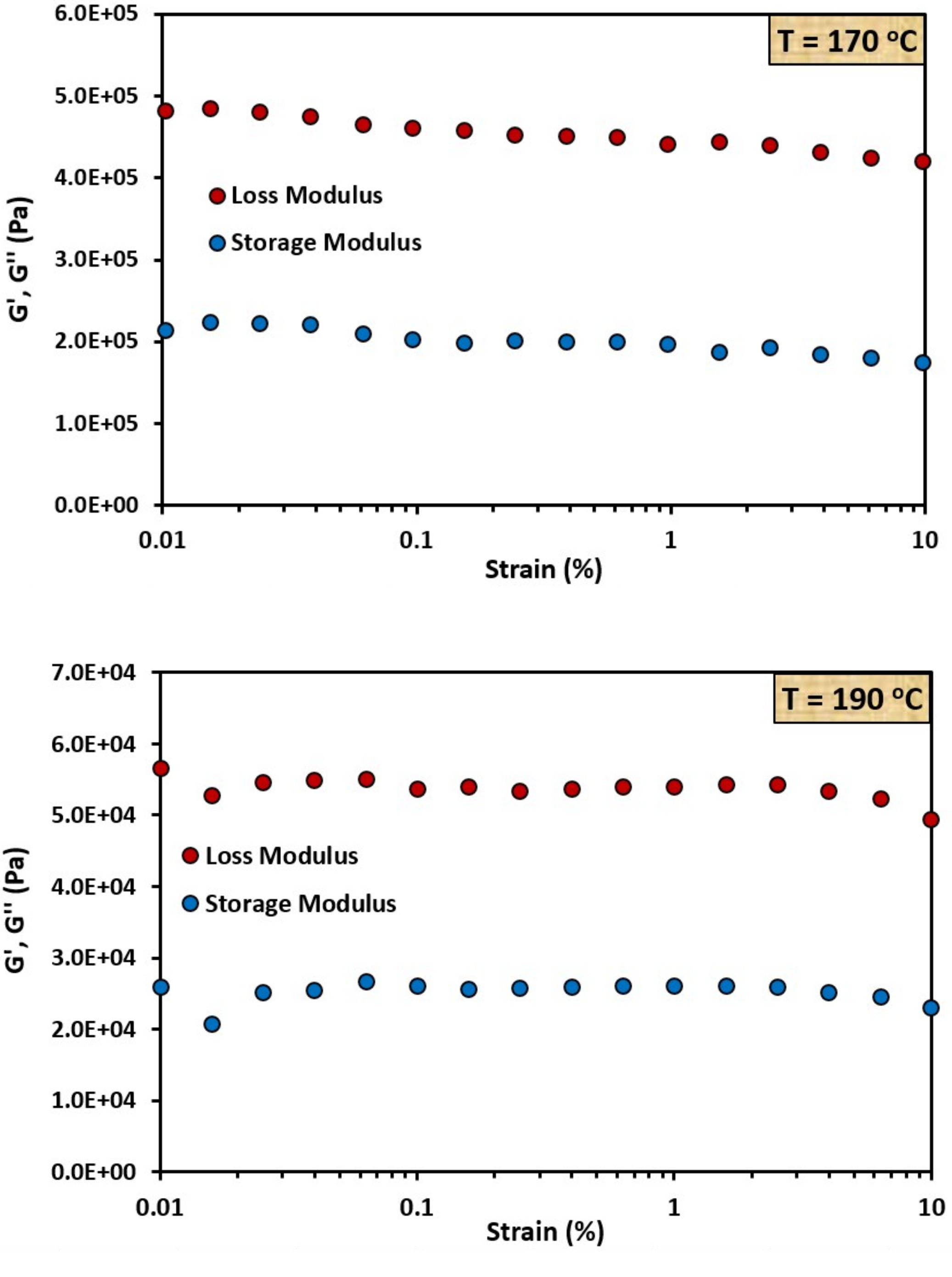

Figure S12. Curves of shear storage modulus (G’) and shear loss modulus (G’’) *versus* strain for *My*-52-IBOMA/S performed by amplitude sweep under the frequency of 0.15 Hz at 170 ºC (top) and 190 ºC (bottom).

I) References

(1) M. Fineman and S. D. Ross, *J. Polym. Sci.*, 1950, 5 (2), 259-262.

(2) F. R. Mayo and F. M. Lewis, *J. Am. Chem. Soc.*, 1944, 66 (9), 1594-1601.

(3) T. Kelen and F. Tüdös, *J. Macromol. Sci., Chem.*, 1975, 9 (1), 1-27.

(4) P. W. Tidwell and G. A. Mortimer, *J. Polym. Sci., Part A: Gen. Pap.*, 1965, 3, 369-387.

(5) P. Sarkar and A. K. Bhowmick, *RSC Adv.*, 2014, 4, 61343-61354.

(6) D. W. van Krevelen and K. Te Nijenhuis, in *Properties of Polymers, 4th edition*, ELSEVIER, 2009, ch. 7, pp. 189-227.
